# 5′-*O*-Methylphosphonate nucleic acids—new modified DNAs that increase the *Escherichia coli* RNase H cleavage rate of hybrid duplexes

**DOI:** 10.1093/nar/gku125

**Published:** 2014-02-12

**Authors:** Hana Šípová, Tomáš Špringer, Dominik Rejman, Ondřej Šimák, Magdalena Petrová, Pavel Novák, Šárka Rosenbergová, Ondřej Páv, Radek Liboska, Ivan Barvík, Josef Štěpánek, Ivan Rosenberg, Jiří Homola

**Affiliations:** ^1^Institute of Photonics and Electronics AS CR, v.v.i., Chaberská 57, 182 51 Prague, Czech Republic, ^2^Institute of Organic Chemistry and Biochemistry AS CR, v.v.i., Flemingovo nám. 2., 166 10 Prague, Czech Republic and ^3^Faculty of Mathematics and Physics, Charles University in Prague, Ke Karlovu 3, 121 16 Prague, Czech Republic

## Abstract

Several oligothymidylates containing various ratios of phosphodiester and isopolar 5′-hydroxyphosphonate, 5′-*O*-methylphosphonate and 3′-*O*-methylphosphonate internucleotide linkages were examined with respect to their hybridization properties with oligoriboadenylates and their ability to induce RNA cleavage by ribonuclease H (RNase H). The results demonstrated that the increasing number of 5′-hydroxyphosphonate or 5′-*O*-methylphosphonate units in antisense oligonucleotides (AOs) significantly stabilizes the heteroduplexes, whereas 3′-*O*-methylphosphonate AOs cause strong destabilization of the heteroduplexes. Only the heteroduplexes with 5′-*O*-methylphosphonate units in the antisense strand exhibited a significant increase in *Escherichia coli* RNase H cleavage activity by up to 3-fold (depending on the ratio of phosphodiester and phosphonate linkages) in comparison with the natural heteroduplex. A similar increase in RNase H cleavage activity was also observed for heteroduplexes composed of miRNA191 and complementary AOs containing 5′-*O*-methylphosphonate units. We propose for this type of AOs, working via the RNase H mechanism, the abbreviation MEPNA (MEthylPhosphonate Nucleic Acid).

## INTRODUCTION

Regulation of gene expression by modified nucleic acids holds great potential for both research purposes and the medical treatment of various diseases (1). The transfer of genetic information from DNA to proteins can be regulated by different types of oligonucleotides, such as antisense oligonucleotides (AOs), ribozymes and double-stranded RNAs in RNA interference. AOs are composed of modified units, which prevent their fast degradation by cell nucleases. They act through two generally accepted mechanisms: (i) the mechanism of steric block, in which the splicing or translation of the target mRNA is physically prevented by hybridized AOs and (ii) the mechanism of ribonuclease H (RNase H), which is associated with mRNA cleavage. RNase H is a ubiquitous endonuclease that hydrolyzes the RNA strand of RNA/DNA hybrid duplexes. The inhibition of gene expression via the RNase H mechanism is an attractive approach with considerable therapeutic potential in the treatment of viral and malignant diseases. An antisense drug has been introduced into the market for treatment of cytomegalovirus-induced retinitis (*Vitravene*, ISIS Pharmaceuticals, USA) (2). Recently an oligonucleotide-based drug inhibitor of apolipoprotein B was approved by US Food and Drug Administration for treatment of homozygous familial hypercholesterolemia (*Kynamro*, ISIS Pharmaceuticals, USA) (3). The efficiency of RNA cleavage by RNase H and subsequent downregulation of the corresponding protein has been demonstrated in several eukaryotic systems (4–6), for review see (1,7,8).

The ability of RNase H to cleave RNA in an AO*RNA heteroduplex is influenced by numerous factors (9). There are structural parameters of AO*RNA heteroduplex that are essential for its recognition as a substrate for RNase H (10): (i) a conformationally flexible sugar with an eastern *O4′-endo* sugar pucker, (ii) a conformationally rigid sugar-phosphate backbone, (iii) duplex conformation between the A and B forms and (iv) an accessible minor groove in the heteroduplex for RNase H binding. Moreover, there are other important parameters for successful AOs, such as resistance to nucleolytic degradation and low nonspecific interactions with cellular proteins. It is the unpredictability of these factors that renders the design of new chemical modifications difficult.

To date, the most successful AOs have been isoelectronic phosphorothioate AOs (11,12). Hybrid duplexes of phosphorothioate AOs with RNA exhibit ∼2-fold lower RNase H cleavage rate than natural heteroduplexes (13). The limitation of phosphorothioate AOs are their nonspecific high-affinity hydrophobic interactions with certain proteins and, at higher concentrations, the inhibition of human RNase H and DNA polymerase (13,14), which can lead to cellular toxicity (15). Other modified oligonucleotides exist that also stimulate RNase H activity, such as phosphorodithioate (16,17), P-chiral boranophosphate (18), cyclohexenyl (CeNA) (19,20) and 2′-fluoro-2′-deoxyarabinofuranosyl (2′F-ANA) (21) AOs. Boranophosphate oligodeoxynucleotides were shown to enhance the RNase H cleavage rate of a poly(rA):oligo(dT) substrate (22), and 2′F-ANA were found to increase duplex stability and induce RNase H activity (21). The cleavage kinetics for 2′F-ANA have not yet been published. However, the 2′F-ANA were found to increase duplex stability and induce RNase H activity, and therefore, they may have considerable therapeutic potential (23–26). CeNA represent oligonucleotides with cyclohexene sugar mimics (19,20). These oligonucleotides show enhanced affinity for RNA targets but fail to stimulate RNase H cleavage activity (their K_cat_ is 500-fold lower than that of phosphodiester oligodeoxynucleotides). Recently, the interesting properties of chimeric phosphonoacetate (ACE) and thiophosphonoacetate (S-ACE) oligodeoxynucleotides were reported. Although the RNase H-mediated cleavage of RNA*AO heteroduplexes containing ‘pure’ ACE or S-ACE AOs is slow, AOs with ACE and S-ACE linkages and phosphorothioate gaps increase the initial cleavage rate of RNase H >2.5-fold in comparison with those of phosphodiester/phosphorothioate heteroduplexes (27,28). Moreover, these chimeras exhibit high nuclease resistance and significantly reduced T_m_ values that increase selectivity in the recognition of mismatch pairs. All aforementioned modified oligonucleotides possess isopolar and isosteric internucleotide linkages.

**Figure 1.**
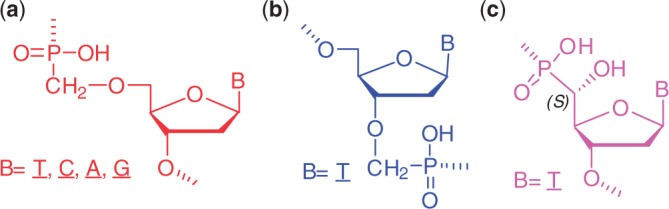
Chemical structures of the nucleoside phosphonate units used in this study: (**a**) 5′*-O-*methylphosphonate, (**b**) 3′-*O*-methylphosphonate and (**c**) 5′-hydroxyphosphonate.

Thus far, only little attention has been devoted to nonisosteric modifications of the internucleotide linkage, i.e. oligonucleotides with longer or shorter linkages in their sugar-phosphate backbones (29,30). Our study focused on three types of isopolar nonisosteric phosphonate-based modifications: (i) a shorter type, 5′-(*S*)-hydroxyphosphonate, and (ii) two longer types, regioisomeric 5′- and 3′-*O*-methylphosphonates (Figure 1), which differ in the position of the extra methylene group in the internucleotide linkage. Using a recently developed surface plasmon resonance (SPR) biosensor-based method (31), we studied the ability of the corresponding hybrid duplexes to elicit RNase H activity with respect to the thermal stability of the duplexes and the stereochemistry of the phosphonate internucleotide linkages.

## MATERIALS AND METHODS

### Chemicals

Carboxylic (HS-C_11_-EG_3_-OCH_2_-COOH) and hydroxylic (HS-C_11_EG-OH) alkanethiols were obtained from Prochimia (Sopot, Poland). Ethanolamine hydrochloride, *N*-hydroxysuccinimide and 1-ethyl-3-(3-dimethylaminopropyl) carbodiimide hydrochloride were purchased from Biacore (Uppsala, Sweden). Streptavidin from *Streptomyces avidinii* and bovine serum albumin (BSA) were purchased from Sigma-Aldrich (St. Louis, MO, USA). RNase H from *E. coli* and RNase H buffer were purchased from Invitrogen (Coralville, IA, USA).

Biotinylated DNA and RNA oligonucleotides [P1 (49-mer): biotin-(TEG)_2_-5′-dC_7_-rA_16_-d(CCA)_8_dC-3′, Pr191: biotin-(TEG)_2_-5′-r(CAA CGG AAU CCC AAA AGC AGC UG)-d(CCA)_8_-dC-3′, BdTG_25:_ biotin-(TEG)_2_-5′-d(GTG)_8_-dG-3′, Pd191: biotin-(TEG)_2_-5′-d(CAA CGG AAT CCC AAA AGC AGC TG)-d(CCA)_8_-dC-3′], natural dT_15_ and microRNA191 [5′-r(CAA CGG AAU CCC AAA AGC AGC UG)-3′] were purchased from Integrated DNA Technologies (USA). All commercially obtained oligonucleotides were high pressure (or high performance) liquid chromatography (HPLC) grade.

AOs containing various ratios of phosphodiester and 5′-*O*-methylphosphonate (Figure 1a), 3′-*O*-methylphosphonate (Figure 1b) and 5′-hydroxyphosphonate (Figure 1c) units were synthesized according to our previously described protocol (32). Their structures are summarized in Tables 1–4.

**Table 1.** Hybridization and RNase H cleavage parameters for the hybrid duplexes containing 5′*-O-*methylphosphonate oligothymidylates and rA_15_

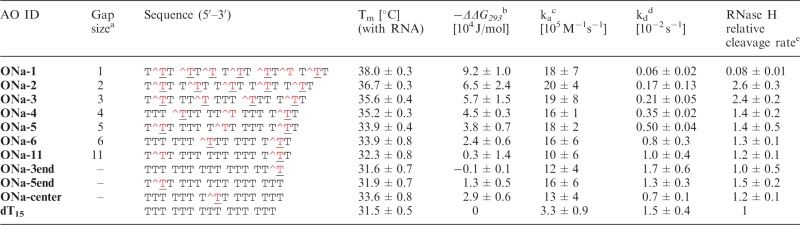

^T denotes the modified unit **a** (Figure 1).

^a^Number of consecutive natural units.

^b^Difference in Gibbs free energy (−ΔΔG_293_) between the modified and natural hybrid duplexes (dT_15_*rA_15_).

^c^Association rate constant.

^d^Dissociation rate constant.

^e^*K*_cat_^AO^/*K*_cat_^dT15^.

**Table 2.** Hybridization and RNase H cleavage parameters for the hybrid duplexes containing 3′-*O*-methylphosphonate oligothymidylates and rA_15_

AO ID	Gap size^a^	Sequence (5′–3′)	T_m_ [°C] (with RNA)	−*ΔΔG_293_*^b^ [10^4^ J/mol]	k_a_^c^ [10^5^ (Ms)^−1^]	k_d_^d^ [10^−2^ s^−1^]	RNase H relative cleavage rate^e^
**ONb-5**	5	T^TT TTT T^TT TTT T^TT	28.3 ± 0.3	−3.6 ± 0.7	3.2 ± 1.1	1.9 ± 0.4	0.46 ± 0.01
**ONb-6**	6	TTT TTT^TTT TTT T^TT	29.0 ± 0.9	−2.8 ± 0.8	3.3 ± 0.9	1.4 ± 0.1	0.49 ± 0.01
**ONb-11**	11	T^TT TTT TTT TTT T^TT	29.5 ± 0.4	−2.1 ± 1.0	3.6 ± 0.2	1.2 ± 0.3	0.60 ± 0.01
**ONb-3end**	–	TTT TTT TTT TTT TT^T	30.8 ± 0.2	−1.4 ± 0.9	2.8 ± 0.5	0.7 ± 0.3	0.74 ± 0.03
**ONb-5end**	–	T^TT TTT TTT TTT TTT	31.4 ± 0.6	−0.5 ± 0.9	3.9 ± 0.8	0.7 ± 0.2	0.52 ± 0.01
**dT_15_**	–	TTT TTT TTT TTT TTT	31.5 ± 0.5	0	3.3 ± 0.9	1.5 ± 0.4	1

T^ denotes the modified unit **b** (Figure 1).

^a^Number of consecutive natural units.

^b^Difference in Gibbs free energy (−ΔΔG_293_) between the modified and natural hybrid duplexes (dT_15_*rA_15_).

^c^Association rate constant.

^d^Dissociation rate constant.

^e^*K*_cat_^AO^/*K*_cat_^dT15^.

**Table 3.** Hybridization and RNase H cleavage parameters for hybrid duplexes containing 5′-hydroxyphosphonate oligothymidylates and rA_15_

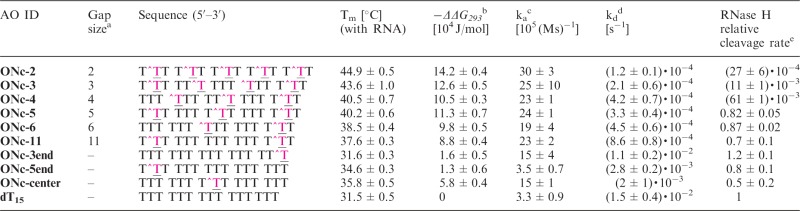

^T denotes the modified unit **c** (Figure 1).

^a^Number of consecutive natural units.

^b^Difference in Gibbs free energy (−*ΔΔG_293_*) between the modified and natural hybrid duplexes (dT_15_*rA_15_).

^c^Association rate constant.

^d^Dissociation rate constant.

^e^*K*_cat_^AO^/*K*_cat_^dT15^.

**Table 4.** Hybridization and RNase H cleavage parameters of MEPNA targeted toward miRNA191

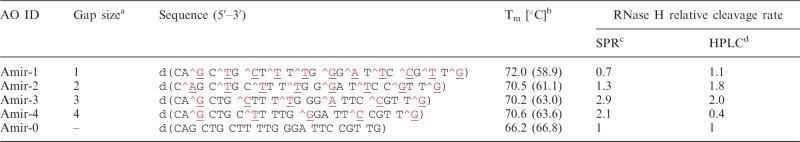

^a^Number of consecutive natural units.

^b^Melting temperature of the Amir*miRNA191 heteroduplex (the numbers in parentheses correspond to the melting temperatures of the duplexes formed by Amir AOs with DNA that was isosequential to miRNA191).

^c^*K*_cat_^AO^/*K*_cat_^natural DNA^

^d^*t*_50_^natural DNA^/*t*_50_^AO^_,_ t_50_ is time in which 50% of the heteroduplexes were cleaved, determined by linear interpolation of the data in Figure S4.

### SPR measurements

In this work, an SPR biosensor with the option of four sensing spots that was developed at the Institute of Photonics and Electronics (Prague, Czech Republic) was used. The sensor is based on the attenuated total reflection method and wavelength spectroscopy of surface plasmons (33). In this sensor, white light is used to excite surface plasmons on a thin gold film in four areas, which are interfaced with four separate chambers of a flow cell (34).

The SPR sensor chip was functionalized with a self-assembled monolayer of mixed alkanethiols containing hydroxyl and carboxyl terminal groups. Streptavidin was covalently attached to the carboxylic groups using amine-coupling chemistry. A detailed description of functionalization procedure is provided in a previous paper from our laboratory (31).

#### SPR study of AO hybridization

All hybridization measurements were performed at 20°C and a flow-rate of 30 µl/min. The surface of the SPR sensor chip with immobilized streptavidin was first equilibrated with phosphate buffered saline buffer (137 mM NaCl, 1.4 mM KH_2_PO_4_, 8 mM Na_2_HPO_4_.12 H_2_O, 2.7 mM KCl, pH 7.4 at 25°C) at a flow rate of 30 µl/min until no baseline change was observed. Then, a 50 nM solution of biotinylated oligonucleotide P1 was pumped over the surface. The reference surface was functionalized with a noncomplementary oligonucleotide (BdTG_25_). Hybridization was then monitored in hybridization buffer (75 mM KCl, 50 mM Tris–HCl, 3 mM MgCl_2_, pH 8.3 at 25°C). A solution containing the AO at a concentration in the range of 5–100 nM was injected into both the measuring and reference channels.

The resultant duplex formation was considered to be a pseudo-first–order process. Reference-compensated sensor responses to at least three concentrations were fitted with the Langmuir model using BiaEvaluation software (Biacore, Upsala, Sweden), taking mass transport into account. At least two sets of concentrations for each AO were measured. The dissociation constant (K_D_), association rate constant (k_a_) and dissociation rate constant (k_d_) were determined as averages of the obtained values. The difference between the change of Gibbs free energy that was associated with duplex formation was determined using the van’t Hoff equation: ΔΔG_293_ = RT ln(K_D_^AO^/K_D_^dT15^).

#### SPR study of RNase H activity

**Figure 2.**
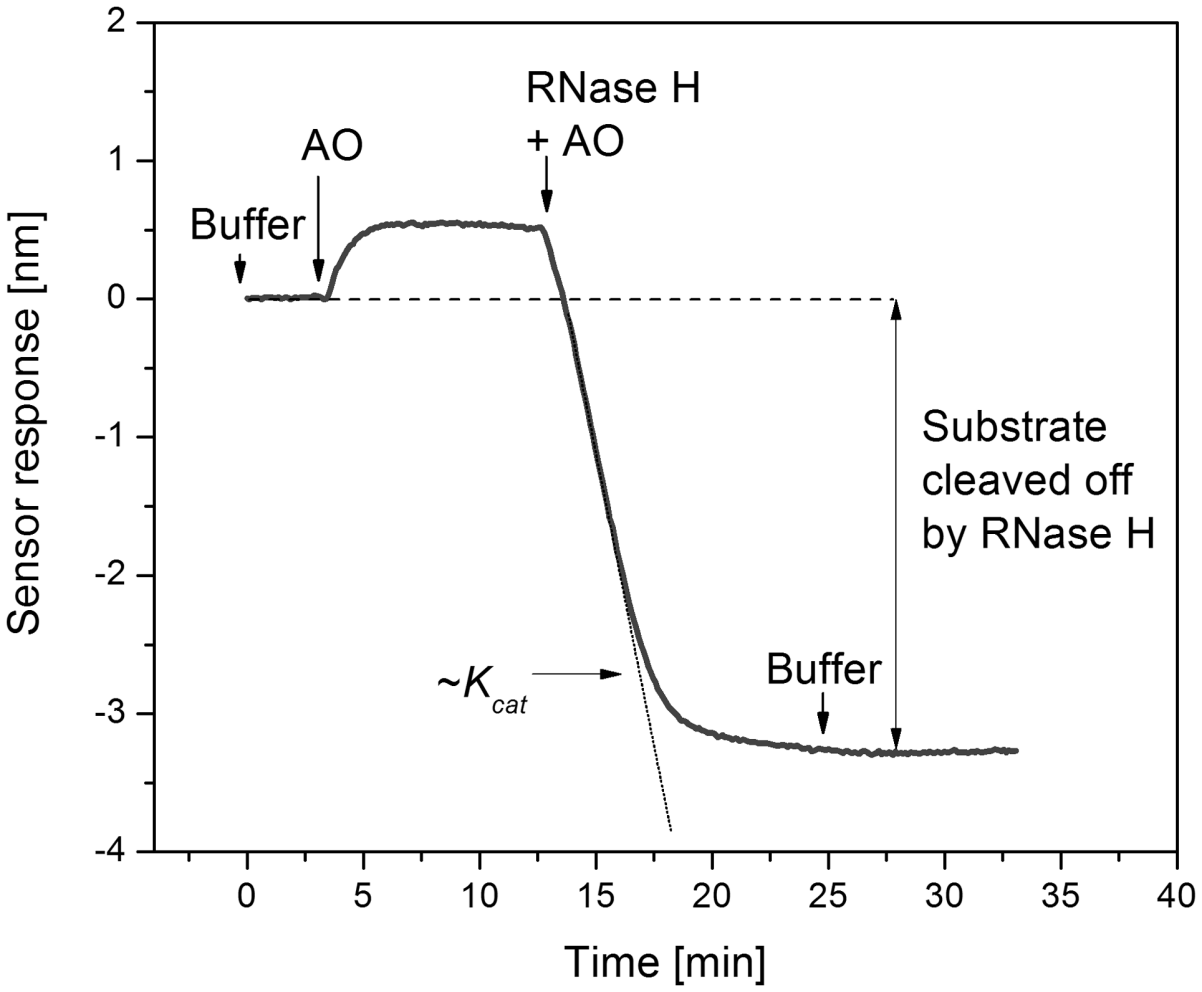
Design of the SPR experiment for the study of RNase H activity. Oligonucleotide immobilization, which gave the reference level (shown as a dashed line), was followed by hybridization of the injected AO and immobilized probe. The solution of RNase H and the AO was injected immediately after the AO hybridization. Hydrolysis of the probe was observed as a decrease in the sensor response. The difference between the initial level and level after the RNase H injection was proportional to the amount of cleaved substrate.

All of the SPR experiments were carried out in a flow-through arrangement at a rate of 20 μl/min and a temperature of 20°C. After immobilization of the P1 probes, the sensor surface was washed for 5 min with reaction buffer (RNS; 75 mM KCl, 50 mM Tris–HCl, 3 mM MgCl_2_, 10 mM dithiothreitol, 2.5% glycerol, pH 8.3 at 25°C). The solution of AO was then pumped through the flow chamber for 10 min, and the formation of probe*AO complexes was monitored (Figure 2). Due to the low stability of some of the RNA*AO hybrid duplexes at the temperatures used, the sensor surface was not washed with buffer but was directly exposed to the solution of RNase H (5 or 2.5 UI/ml) and an AO at the same concentration as in the previous step in reaction buffer. The RNase H-mediated hydrolysis of the immobilized probe was observed as a decrease in the sensor response, as the mass of the bound molecules was reduced (Figure 2). After 10–15 min, the RNS buffer was pumped through the flow chamber again. The difference between the baseline in the RNS buffer before incubation with the solution of AO and after washing with buffer equaled the amount of cleaved probe. The *K_cat_* was determined as the maximal negative slope of the sensor response to P1 or Pr191 cleavage with RNase H.

### UV-Vis melting studies

UV absorption measurements were performed in the buffer used in the SPR experiments (i.e. 10 mM HEPES, 75 mM KCl, 3 mM MgCl_2_, pH 8.3 at 25°C) for the oligothymidylates and in modified buffer (10 mM HEPES, 75 mM KCl, 0.1 mM EDTA, pH 8.3 at 25°C) for the oligonucleotides that were antisense to miR191. Equimolar solutions of mixed oligonucleotides with a total strand concentration of 2 µM were placed in cuvettes with a 1-cm path length. The absorbance at the wavelength of 260 nm was measured with a Varian 4000 UV-VIS spectrophotometer (Agilent Technologies, Santa Clara, CA, USA). Heating and cooling cycles were executed at a temperature range of 30–90°C and a rate of 1°C/min. Each sample was measured in duplicate. The melting temperatures were calculated by the standard derivative method.

### HPLC measurements

The enzymatic reaction was studied at 25°C in solution containing 1.4 µM AO, 1.3 µM miR191 and 20 UI/ml RNase H *E. coli* in reaction buffer (44 mM Tris–HCl, 4.4 mM MgCl_2_, 80 mM KCl, 8 mM DTT, 10% glycerol, 10 µg/ml BSA, pH 8.3 at 25°C). The reaction was stopped by addition of EDTA at 40 mM concentration. The high-resolution anion-exchange chromatography of oligonucleotides and the products of the RNase H-mediated cleavage was performed on DNAPac Dionex PA100 column (4 × 250 mm; 1 ml/min, 55°C) using a concave gradient (No. 5) of sodium chloride (20 mM → 1.5 M) in 20 mM sodium acetate buffer (pH 7.5) generated by Alliance (Waters) HPLC during 30 min. The individual peaks were collected, desalted on C18 Zip-Tips and analyzed by Reflex IV MALDI TOF MS (Bruker Daltonics) using 3-hydroxypyridine-2-carboxylic acid—pyridine-2-carboxylic acid mixture as a matrix.

### Molecular dynamics simulations

*Escherichia coli* RNase H + rA_10_*dT_10_ complex was derived from our previous model (35) by insertion of the second magnesium ion into the *E. coli* RNase H active site [in accordance with the crystal structure of ‘Human’ RNase H (36)]. The binuclear active site of *E. coli* RNase H was stabilized in molecular dynamics simulations (MDS) by means of polarization of the scissile phosphate group of RNA as described previously (37). The 5′(R/S)-hydroxyphosphonate, 3′/5′*-O-*methylphosphonate internucleotide linkages and 2′*-O-*methyl nucleotides were modeled as described in our previous studies (30,37–39). For details of MDS methodology, see the Supplementary Material.

## RESULTS

### Influence of the modification number and position on the stability of the AO-RNA complex

**Figure 3.**
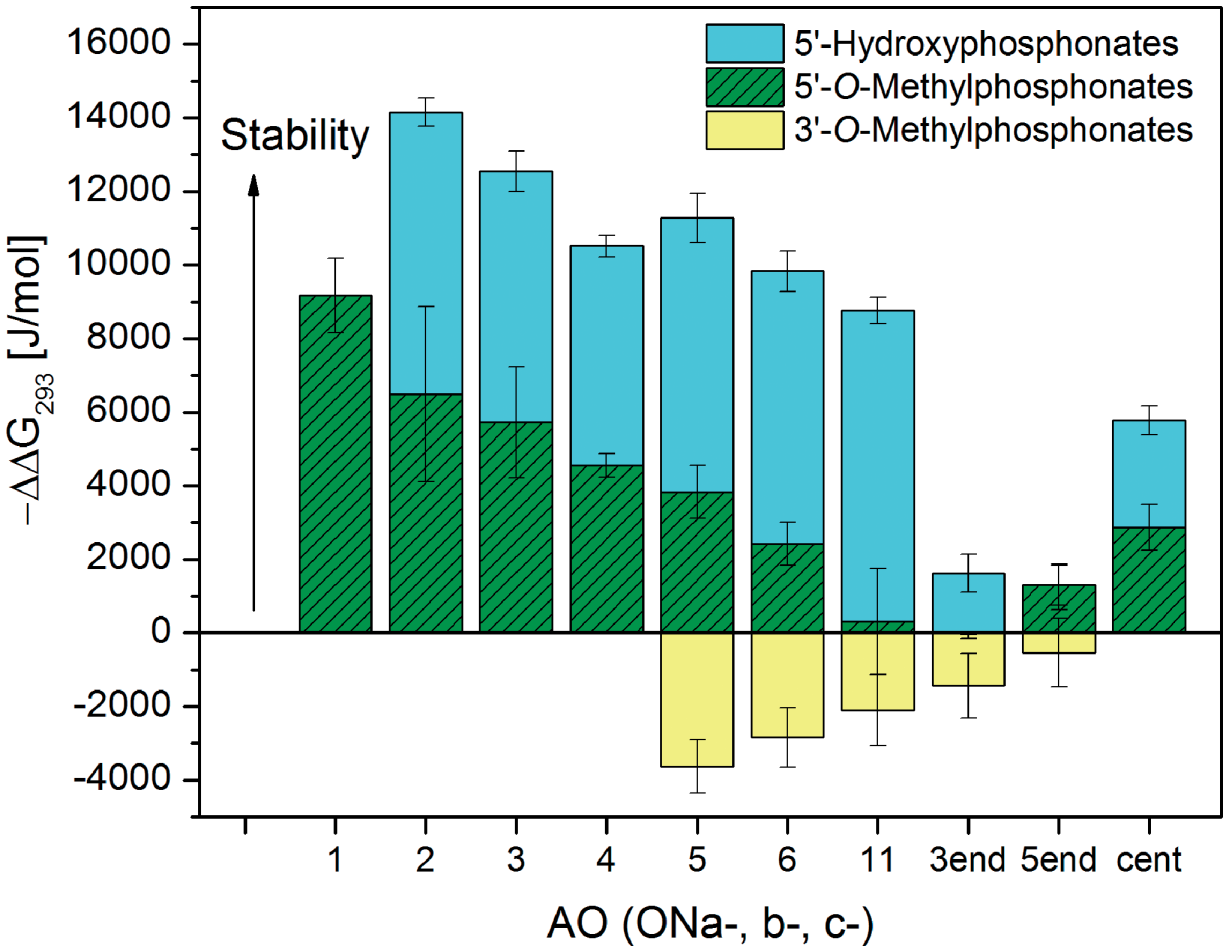
Differences in the Gibbs free energy −ΔΔG_293_ between the hybrid duplexes AO*rA_15_ and dT_15_*rA_15_ obtained from the SPR measurements. The x-axis denotes the size of the gap containing unmodified units or the position of a single modification. The −ΔΔG_293_ values for the 5′*-O-*methylphosphonate and 5′-hydroxyphosphonate modifications at the 5′-end position are equal. The exact positions of the modifications are listed in Tables 1–3.

Tables 1–3 and Figure 1 summarize the sequences and structures, respectively, of the modified mixed-backbone oligothymidylates (dT_15_) used in this study as model AOs. The AOs contained phosphonate internucleotide linkages alternating with gaps of unmodified consecutive phosphodiester linkages. Hybridization of the oligothymidylate to a probe containing rA_16_ was studied with an SPR sensor (data shown in Supplementary Figure S1). The kinetic curves were fitted with a 1:1 Langmuir model to obtain the association and dissociation rate constants. The Gibbs free energy of the AO*rA_15_ duplexes was calculated from the equilibrium dissociation constant as described in the ‘Materials and Methods’ section, and it was compared with the Gibbs free energy of the unmodified dT_15_*rA_15_ duplex. Figure 3 shows the relative increase or decrease in the Gibbs free energy of the heteroduplex caused by the modifications at different positions.

Tables 1–3 present the determined hybridization and RNase H cleavage parameters for the different modification types analyzed in this study. Both equilibrium parameters that characterize the stability of the duplexes, i.e. the melting temperature obtained from the UV melting curves measured in solution and the difference in the Gibbs free energy obtained from the SPR experiments, correlate perfectly (with an unimportant exception of **ONc-5end**). This proves that the conclusions drawn from the SPR results are also valid for oligonucleotide interactions in solution.

Table 1 (and Figure 3) shows that the 5′*-O-*methylphosphonate modifications (see Figure 1 for the chemical structure) increased the stability of the hybrid duplexes. The observed stability increase (see Table 2 for the numerical values) grew with the number of modifications in the AO backbone but also depended on the size of the tract of natural thymidylates in the sequence. A large unmodified segment reduced the stability effect of the modification. It was demonstrated, e.g. by the lower stability of the duplex formed by **ONa-11** in comparison with **ONa-6** (both of which contained two modified linkages). On the other hand, a single modification situated in the center of the AO (**ONa-center**) led to stability that was comparable with that of AOs with two modified linkages (**ONa-11** and **ONa-6**). We also observed an impact of the modification position on the stability of the hybrid duplex, which mainly concerned the end sites. The introduction of the 5′-*O*-methylphosphonate unit to the 5′-end (**ONa-5end**) provided a stabilizing effect, whereas the 3′-end modification of the AO (**ONa-3end**) had no effect on the heteroduplex stability.

Despite the large similarity between 5′-*O-* and 3′-*O*-methylphosphonate internucleotide linkages, which differ only in the position of the bridging CH_2_ group with respect to the phosphorus atom (see Figure 1 for the chemical structure), the introduction of 3′-*O*-methylphosphonate units decreased the duplex stability (Figure 3 and Table 2) in contrast to the 5′-*O*-methylphosphonate units. The destabilization effect of the 3′-*O*-methylphosphonate units exhibited analogous trend as observed for the stabilization effect of the 5′-units. First, destabilization increased with the number of modifications but was reduced with a larger tract of natural thymidylates (compare the data for **ONb-6** and **ONb-11**). Second, while the placement of the 3′-*O*-methylphosphonate unit at the 3′-end (**ONb-3end**) resulted in destabilization as observed for **ONb** with several modifications, its placement at the 5′-end (**ONb-5end**) had no effect on the heteroduplex stability.

5′-Hydroxyphosphonate modifications, in which a shortened internucleotide linkage is present compared with unmodified nucleotides (see Figure 1 for the chemical structure), significantly stabilized the hybrid duplexes (Figure 3). In terms of the Gibbs free energy, the stabilization efficiency was ∼2-fold higher than that for 5′-hydroxyphosphonate AOs (Table 3). The average ΔT_m_ per nucleotide unit was +3°C when this modification was alternated with phosphodiester bonds in a 1:2 ratio (**ONc-2**), i.e. between the reported ΔT_m_ values for LNA- and 2′-*O*-methyl-substituted RNA oligonucleotides [+9 and +2°C, respectively (20)]. Unlike the 5′-*O*-methylphosphonate AOs (MEPNA), the stabilization effect of the 5′-hydroxyphosphonate modifications did not seem to depend on the size of the unmodified segment but solely on the number of modifications. With up to five modifications (**ONc-3**), the increase in the T_m_ was approximately proportional to the modification number. The effect of the modification position at the end of the chain was analogous to the 5′-*O*-methylphosphonate modification; the ΔT_m_ of the duplex formed by **ONc-5end** was similar to that of **ONc-center** and was close to the line of the proportionality between the ΔT_m_ and the number of modifications, while modification at the 3′ end (**ONc-3end**) did not influence the T_m_ value.

**Figure 4.**
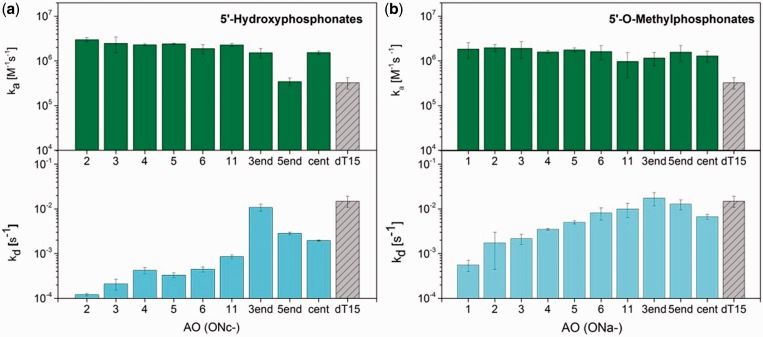
Association and dissociation rate constants of AO*rA15 heteroduplex formation. (**a**) AOs with 5′-hydroxyphosphonate linkages. (**b**) AOs with 5′-*O*-methylphosphonate linkages.

Figure 4 displays the association and dissociation rate constants for the hybrid duplexes of rA_15_ with AOs containing 5′-*O*-methylphosphonate and 5′-hydroxyphosphonate modifications. The ratio of these rates represents the equilibrium constant (the decrease in the Gibbs free energy on duplex formation is proportional to the difference of their logarithms). The rate constant values can therefore highlight which of the two processes—duplex formation or its dissociation—is affected by the oligothymidylate modification. As shown in Figure 4, the modifications influenced both processes but in a different way.

The association rate constant was increased 4- to 9-fold when any two modifications were introduced, with exception of **ONc-5end**, where the association rate constant remained unchanged. The increase in the association rate constant indicates a lowering of the activation energy on duplex formation. It is interesting that this increase (with the abovementioned exception) did not depend on the modification position and grew only little with the number of modifications. The effect of the 5′-hydroxyphosphonate modifications was slightly higher than that of the 5′-*O*-methylphosphonate modifications.

The dominating contribution to the equilibrium duplex occurrence comes, however, from the decrease in the dissociation rate constant; in the case of **ONc-2**, this value is 100-fold lower than that of the unmodified dT_15_*rA_15_ duplex. This finding demonstrates that the two modifications mainly support the thermodynamic stability of the hybrid duplex, in which the 5′-hydroxyphosphonate modification is more effective than the 5′-*O*-methylphosphonate modification. For both modification types, the dissociation rate constant decreased with the number of modifications. The exceptions to this trend are single modifications placed at the 3′-end of the AO chain (**ONa-3end** and **ONc-3end**) that have no effect and the single 5′-*O*-methylphosphonate modification in the center of the AO chain (**ONa-center**) that has a stronger effect. The latter demonstrates the importance of the length of the unmodified thymidylate tract on AO stability in the case of 5′-*O*-methylphosphonate modifications. In contrast to the two aforementioned modifications, the introduction of the 3′-*O*-methylphosphonate modification influenced both rate constants only weakly (Table 2).

### Influence of modification number and position on RNase H activity

An SPR biosensor was used to determine the kinetics of the AO-induced hydrolysis of the RNA strand in the AO* rA_15_ hybrid duplex by RNase H. The probe **P1**, which contained the rA_16_ sequence, was immobilized on the surface of the SPR sensor. A typical sensor response for the AO hybridization to **P1** and the RNase H hydrolysis of **P1** is shown in Figure 2. The RNase H cleavage rate *K_cat_* was measured as the maximal negative derivative of the sensor response during surface incubation with RNase H. The ratios of the maximal derivatives (*K_cat_^AO^/K_cat_^dT^^15^*) for the AOs containing 5′-*O*-methylphosphonate, 5′-hydroxyphosphonate and 3′-*O*-methylphosphonate units are summarized in Tables 1–3. The curves of the SPR sensor responses are provided in the Supplementary Material (Supplementary Figure S2).

The 5′-hydroxyphosphonate modifications, which remarkably stabilized the AO*rA_15_ duplexes, decreased the RNase H activity (see Table 3) depending on the size of the tract of unmodified thymidylates. If the gap between the modifications included at least 5 nt, the cleavage rate was decreased only slightly (the factor varied from 0.7 to 0.9). The exceptions were **ONc-center**, which decreased the activity of the enzyme by half, suggesting that the position of the unmodified segment is also important, and **ONc-3end**, which contained one modification at the 3′-end and slightly increased the RNase H activity. The RNase H activity significantly dropped for AOs with gap sizes smaller than five (**ONc-4**) and further decreased with decreasing gap size (**ONc-3** and **ONc-2**). Further increasing the number of 5′-hydroxyphosphonate units in the AOs to a ratio of 1:1 completely blocked RNA cleavage (data not shown). This finding is in agreement with previous studies reporting that the *E. coli* RNase H requires at least five consecutive natural nucleotide units for its activity (40).

The AOs containing 3′*-O-*methylphosphonate units also decreased the RNase H activity (Table 2). This effect was more pronounced for the AOs with a higher number of modifications, which might correlate with the decreased stability of the hybrid duplex. Only the AO containing a single 3′-*O*-methylphosphonate unit positioned at the 5′-end (**ONb-5end**) deviated from this trend, as it significantly reduced the RNase H activity. All of the tested AOs with 3′-*O*-methylphosphonate modifications contained a succession of at least five unmodified thymidylates; thus, the dramatic decrease in the RNase H cleavage activity for gaps smaller than five was not seen.

The behavior of the hybrid duplexes containing AOs with 5′*-O-*methylphosphonate units was exceptional. As shown in Table 1, the RNase H cleavage rate of the hybrid duplexes increased with the number of modifications in the AOs; **ONa-2**, with a gap size of two nucleotides, showed an increased cleavage rate of 2.6. Clearly, RNase H activity is promoted by the presence of 5′-*O*-methylphosphonate modifications. **ONa-1**, with a gap size of one, showed a reduced RNA cleavage rate by one order of magnitude compared with dT_15_. As the slightly higher stability of the duplex formed by **ONa-1** compared with **ONa-2** does not seem to be the reason for the low RNase H cleavage activity, the structure of the hybrid duplex with a gap between 5′-*O*-methylphosphonate modifications of <2 nt may not be favorable for RNase H activity.

### MEPNA for miR191

The oligothymidylates with 5′-*O*-methylphosphonate units showed both high affinity to RNA and an ability to activate RNase H, i.e. promising properties for AOs operating via an RNase H mechanism. Therefore, we focused on the 5′-*O*-methylphosphonate modification for the remainder of this study. MicroRNA-191 (miR191) was used as a model RNA target. MiR191, a RNA sequence of 23 nt, is overexpressed in several types of cancer and is a prospective target for cancer gene inhibitors (41).

Four MEPNA for miR191 were prepared with the number of consecutive unmodified nucleotide units (gap size) varying from 1 to 4 (**Amir-1–Amir-4**; **Amir-0** is an unmodified AO). The melting temperatures of the Amir*miR191 duplexes are shown in Table 4. The thermal stability of these duplexes followed the same trend as the 5′-*O*-methylphosphonate oligothymidylates, i.e. it increased with the number of 5′-*O*-methylphosphonate units. **Amir-1**, with a 1:1 ratio of modified and unmodified units, showed the maximal increase in the T_m_ value (+6°C) relative to the unmodified hybrid duplex (**Amir-0**). Table 4 also shows the T_m_ values for duplexes of AOs with DNA that was isosequential to miRNA191. The T_m_ of the natural **Amir-0** duplex with a complementary DNA strand was about the same as for its duplex with RNA. However, the thermal stability of the complex with DNA decreased with an increasing number of 5′-*O*-methylphosphonate units in the AO. For **Amir-1**, the difference between the T_m_ values for its complexes with RNA and DNA reached 13°C. Therefore, it can be concluded that the introduction of 5′-*O*-methylphosphonate units increases not only the stability of AO*RNA duplexes but also the partial selectivity of the AO for the RNA target.

**Figure 5.**
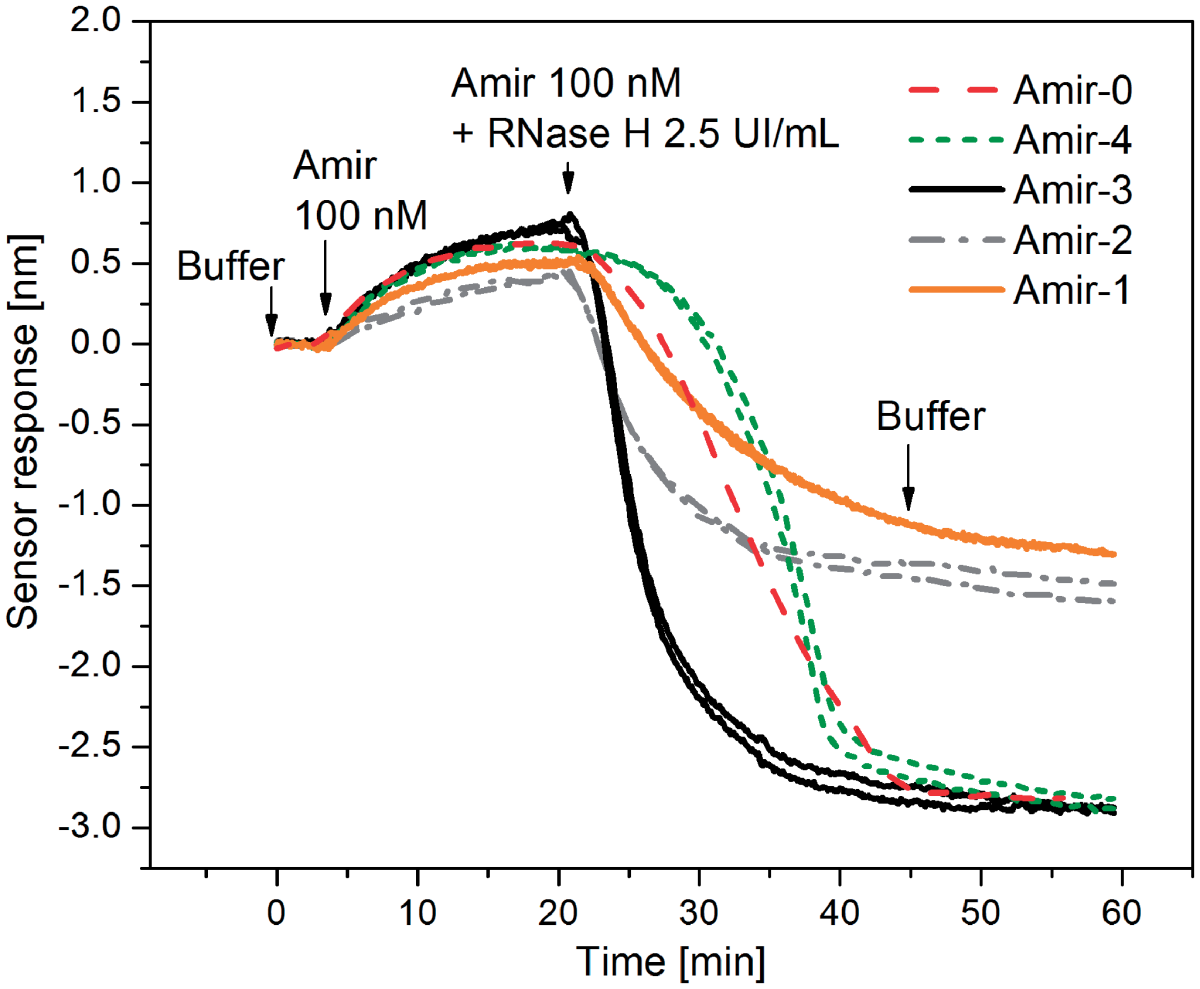
SPR measurements of the RNase H activity on the Amir*miR-191 complex. The sensor response to the hybridization of Amir to the immobilized Pr191 probe containing the miR-191 RNA sequence and the resulting Pr191 cleavage by RNase H is shown. Arrows indicate the injection of the respective solutions.

The biotinylated probe **Pr191**, which contains the miR-191 RNA sequence and a 3′-terminal deoxyribonucleotide sequence to increase its mass that is removed on cleavage by RNase H, was used in the SPR study of RNase H activity. Figure 5 shows the sensor responses to the hybridization of the AOs to the miR-191 sequence in Pr191 and the following cleavage of Pr191 by RNase H.

As shown in Figure 5, all of the AOs (**Amir-1, 2, 3, 4**) were able to activate RNase H. However, the enzyme kinetics showed a complex profile, partially owing to the fact that RNase H might prefer certain positions in the heterogeneous sequence and also that the length of the target RNA (23-mer) allowed for repeated cleavage (the cleaved product can still form a stable heteroduplex with the AO). In particular, the terminal decrease in the sensor response to miR191 cleavage for both **Amir-1** and **Amir-2** was significantly smaller than the decrease observed for native **Amir-0**. This finding suggests that the modifications in **Amir-1** and **Amir-2** cause a shift in the preferred cleavage site to the 3′-end of the miR191 sequence, and therefore, more than half of miR191 remains intact. **Amir-3** induced the full hydrolysis of miR-191. The amount of cleaved miR-191 was similar to that induced with **Amir-0**, but the hydrolysis of miR-191 proceeded at a significantly higher rate.

The kinetics of RNase H action on the duplex formed with **Amir-4** showed a complex profile, as the reaction seemed to proceed at a slower rate initially and at faster rate at the end of the reaction time. Because the SPR method monitors the molecular mass adsorbed to the sensor surface, this complex kinetic profile may have resulted from the superposition of two processes: (i) the adsorption and dissociation of RNase H and (ii) the cleavage of miR-191. Because other AOs did not show such complex profiles, it is probable that the first process was fast, and therefore, the measured kinetics predominantly reflected the kinetics of the RNA cleavage step. It is likely that RNase H tends to associate to the Amir-4*Pr191 duplex for a prolonged time compared with the native heteroduplex and then dissociates after cleavage occurs. However, similar to **Amir-3** and native **Amir-0**, **Amir-4** showed full hydrolysis of miR-191, and the time required for full hydrolysis was similar to native **Amir-0**.

Raw estimates of the K_cat_^AO^ of the RNase H cleavage are displayed in Table 4. As in the case of modified oligothymidylate AOs, these estimates were determined as the negative slopes of the SPR sensor response. The relative cleavage rate was calculated as the ratio of the K_cat_^AO^ obtained for the MEPNA to that of the unmodified deoxyribonucleotide (**Amir-0**). The maximal RNase H cleavage rate was found to be 2.9- and 2.1-fold faster for **Amir-3** and **Amir-4**, respectively, than for the unmodified **Amir-0**. These values correspond well to those obtained for the modified dT_15_ oligonucleotides **ONa-2** and **ONa-3**, with comparable but smaller gap sizes of 2 and 3. On the other hand, although the 5′-*O*-methylphosphonate oligothymidylate **ONa-1**, with a gap size of 1, showed reduced RNase H activity of >10-fold, the K_cat_ for the Amir-1*Pr191 duplex was only slightly lower than that of the unmodified Amir-0*Pr191 duplex.

**Figure 6.**
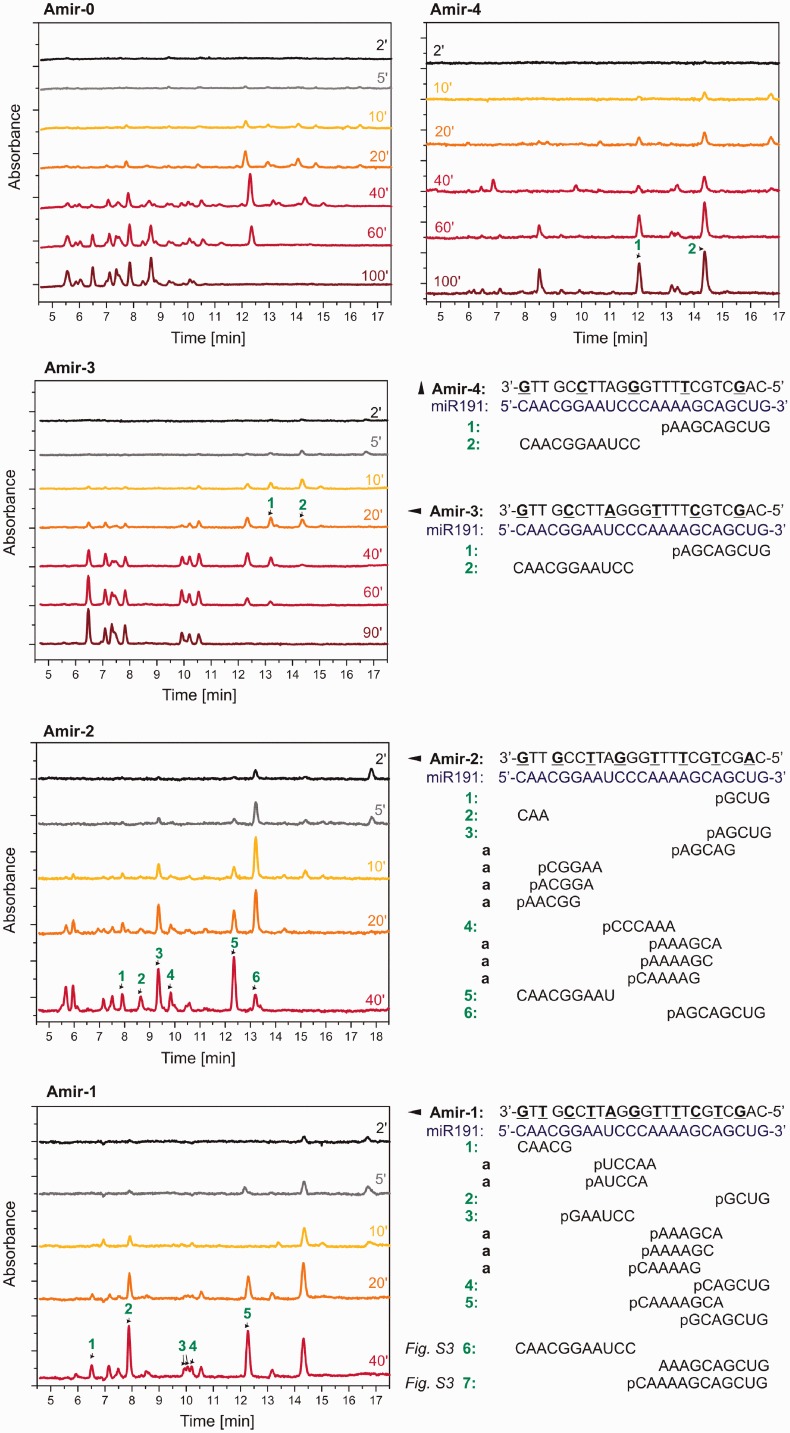
HPLC separation of products of miR191 cleavage by RNase H *E. coli*. miR191 was hybridized with various AOs (Amir-0–Amir-4). The enzymatic reaction was stopped at specific times by the addition of EDTA to the solution. The numbers above selected peaks indicate products identified with MALDI TOF spectroscopy: ‘a’ indicates fragments of the same molecular mass, and any combination of them is possible. Sections of chromatograms corresponding to Amir and hybrid duplexes are shown in Supplementary materials (Supplementary Figure S3).

The results were compared with solution-phase measurements of the RNase H kinetics. First, the RNase H was added to a solution of Amir*miR191 duplexes. The enzymatic reaction was stopped with EDTA at various times. The reaction mixture was then separated by high-resolution anion-exchange chromatography and analyzed with mass spectrometry to identify major components of miR191 cleavage. Supplementary Figure S3 shows a portion of the chromatogram with peaks corresponding to both Amir*miR191 duplexes and free Amir. Over the course of the reaction, it can be seen that the peaks corresponding to the hybrid duplexes and free AO decrease and increase in size, respectively. Furthermore, there are a variety of miR191 fragments hybridized with Amir that were produced. Figure 6 shows fragments of miR191 separated with high-resolution anion-exchange chromatography. The chromatogram of **Amir-0** shows an abundance of various products, starting from long fragments at the beginning of the reaction and ending with tri-, di- and mono-nucleotides at the end of the reaction. It should be noted that these short products are mainly eluted before the 8-min mark. In general, the 5′-*O*-methylphosphonate modifications shifted the preference of RNase H to certain cleavage sites and noticeably decreased the variability of the products (42). Reduction of the number of cleavage sites is the most noticeable in Amir-4*miR191, which showed only two major cleavage sites and most likely represents the reason for the slower cleavage kinetics when compared with **Amir-0** (Supplementary Figure S4). Surprisingly, in this case RNase H did not further cleave the 9-mer and 11-mer fragments of miR191. The variability of products starts to increase with decreasing gap size in the AO, and the **Amir-2** showed a cleavage pattern that was most similar to **Amir-0**. Interestingly, except for fragment 1 in the cleavage induced by **Amir-2**, the miR191 was not cleaved at phosphate locations directly opposite the 5′-*O*-methylphosphonate modification. On the contrary, miR191 in Amir-1*miR191 was dominantly cleaved opposite the 5′-*O*-methylphosphonate modifications.

Supplementary Figure S4 shows the cleavage kinetics determined from concentrations of intact hybrid duplexes by HPLC. Table 4 displays the relative cleavage rates determined from Supplementary Figure S4 as *t*_50_^natural DNA^/*t*_50_^AO^ (*t*_50_ denotes the time in which 50% of the heteroduplexes are cleaved). **Amir-3** and **Amir-2** were determined to increase the cleavage rate in comparison with natural **Amir-0**, while the **Amir-1** cleavage rate was similar to **Amir-0**. Both of these results agree well with the SPR data. The only considerable difference between the results obtained by the solid-phase method (SPR) and solution-phase method (HPLC) was found for **Amir-4**, which exhibited complex interaction behavior as suggested by an unusual kinetics (see Figure 5). These results suggest that AOs with a high abundance of 5′-*O*-methylphosphonate units might be good candidates for antisense compounds owing to their ability to significantly increase the RNase H cleavage rate as well as their sufficient affinity and specificity to the complementary RNA chain.

## DISCUSSION

Three types of isoelectronic nonisosteric DNA modifications were analyzed in this study: 5′-*O*-methylphosphonate, 3′-*O*-methylphosphonate and 5′-hydroxyphosphonate. The incorporation of 5′-*O*-methylphosphonate and 3′-*O*-methylphosphonate units gives rise to ‘lengthened’ regioisomeric *C3*′*-O-P-CH_2_-O-C5*′ and *C3*′*-O-CH_2_-P-O-C5*′ internucleotide linkages, respectively, which differ only in the position of the extra methylene group (structures **a** and **b** in Figure 1). The incorporation of a 5′-hydroxyphosphonate unit provides a shortened *C3*′*-O-P-(HO)C5*′ linkage, with the stereogenic center on the 5′-carbon atom of the modified unit. In the case of 5′(S)-hydroxyphosphonate (structure **c** in Figure 1), a highly populated *trans ****P****-C5*′*-C4*′*-****C3***′ rotamer (43) results in a preorganized structure and high affinity to RNA. In contrast to the 5′(*S*)-epimer, the 5′(*R*)-epimer, which is conformationally promiscuous (34), strongly destabilizes the hybrid helical structures (see also results of MDS in the Supplementary materials, Supplementary Figures S5–S8). Therefore, the 5′(*R*)-epimer was not included in this study.

Introduction of the bridging methylene group into the phosphodiester linkage increases the total entropy of the system by increasing the number of degrees of freedom by 1 per modified unit. The heteroduplexes containing 3′-*O-*methylphosphonate or MEPNA should, therefore, exhibit significantly reduced thermal stabilities in comparison with the unmodified heteroduplex. Surprisingly, only the AOs with 3′-*O-*methylphosphonate units followed this trend. Introduction of 5′-*O-*methylphosphonate units progressively increased the stability of the AO*RNA duplexes, which seems to be rooted in heightened populations of *3*′*-endo* conformers in AO (preliminary results from Raman spectroscopy and MDS—data not shown).

Our study of various gap sizes with **ONb** and **ONc** confirmed that *E. coli* RNase H tolerates modifications that are positioned at least five phosphodiester bonds apart. It corresponds to a portion of AO between the phosphate group bound to Trp85 and the phosphate group opposite to the scissile phosphate of RNA (see the Supplementary materials, Supplementary Figure S8). The 5′-hydroxyphosphonate AOs with four or less consecutive phosphodiester linkages dramatically decreased the RNase H activity (by a factor of 10^2^–10^3^) compared with the unmodified oligonucleotides, suggesting that the conformational rigidity of the duplex prevents interaction with RNase H. MDS showed that 5′-hydroxyphosphonate groups are able to form surprisingly stable contacts with Thr43 and Trp85 amino acids in both DNA binding sites; however, they ultimately led to breakage of four consecutive base pairs (Supplementary materials, Supplementary Figures S9, S10).

There is a significant difference between the regioisomeric 3′- and 5′-*O*-methylphosphonate internucleotide linkages. The negative charge of the phosphonate moiety is shifted along the linkage due to different position of the methylene group. In MDS, the 5′-*O*-methylphosphonate internucleotide linkages were able to form normal contacts with Trp85 and Thr43 amino acids (Supplementary materials, Supplementary Figures S9–S11). In contrast, the methylene group in 3′-*O*-methylphosphonates completely hindered interactions of nonbridging oxygen atoms with Thr43; contacts with Trp85 were lost as well within molecular dynamics runs. Furthermore, the 3′-*O*-methylenephosphonate internucleotide linkage positioned in AO opposite to the scissile phosphate of RNA seems to cause breakage of several consecutive base pairs.

The *E. coli* RNase HI displays strong homology in its amino acid sequence to the catalytic domain to human RNase H1, but it lacks the N-terminal hybrid binding domain that is frequently present in eukaryotes, which enhances the binding affinity of the enzyme for the heteroduplex substrate and increases the positional preference for cleavage [for review, see (44)] (45). The *E. coli* RNase HI therefore lacks significant sequence preference (13). Our analysis of AOs targeting miRNA191 showed that the MEPNA may alter the positional preference of RNase HI as well as the time that the enzyme spends in complex with the substrate. Despite these distinctions, the cleavage rates for MEPNA with 2- to 3-nt gaps were 2-fold higher than the unmodified AO. The high nuclease resistance of the phosphonate *-O-P-CH_2_-O*- bond (29) further increases the potential of the MEPNA as AOs that act via RNase H mechanism.

## CONCLUSIONS

In this study, we analyzed the influence of the position and number of 5′-hydroxyphosphonate, 5′-*O*-methylphosphonate and 3′-*O*-methylphosphonate units in AOs on their affinity to an RNA target and their ability to induce RNase H activity. The 3′-*O-*methylphosphonate AOs significantly destabilized the AO*RNA heteroduplexes and failed to stimulate RNase H activity. The AOs containing MEPNA alternating with two, three or four phosphodiester linkages showed, in heteroduplexes, a superior enhancement of the RNase H cleavage rate up to 3-fold (compared with the unmodified heteroduplex), suggesting the antisense potential of MEPNA. Moreover, MEPNA increased the thermodynamic stability of duplexes with RNA and decreased the stability of duplexes with complementary DNA.

## SUPPLEMENTARY DATA

Supplementary Data are available at NAR Online.

## FUNDING

Praemium Academiae of the Academy of Sciences of the Czech Republic; Czech Science Foundation [203/09/0820 and 13-26526S]; IPE and IOCB institutional research projects [RVO:67985882 and RVO:61388963, respectively]. Funding for open access charge: Czech Science Foundation [13-26526S].

*Conflict of interest statement*. None declared.

## Supplementary Material

Supplementary Data
