# Supplementary material for: 5′-O-Methylphosphonate nucleic acids—new modified DNAs that increase the Escherichia coli RNase H cleavage rate of hybrid duplexes
Source: Nucleic Acids Res. 2014 Feb 12;42(8):5378–89. doi: 10.1093/nar/gku125 (PMC4005664; doi:10.1093/nar/gku125)
Supplement: Supplementary Data [file supp_42_8_5378__index.html]

5′-O-Methylphosphonate nucleic acids—new modified DNAs that increase the Escherichia coli RNase H cleavage rate of hybrid duplexes — 5′-O-Methylphosphonate nucleic acids—new modified DNAs that increase the Escherichia coli RNase H cleavage rate of hybrid duplexes — Supplementary Data 

# 5′-*O*-Methylphosphonate nucleic acids—new modified DNAs that increase the *Escherichia coli* RNase H cleavage rate of hybrid duplexes

## Supplementary Data

files

**Files in this Data Supplement:**

- Supplementary Data - pdf file
