# Supplementary material for: 5′-O-Methylphosphonate nucleic acids—new modified DNAs that increase the Escherichia coli RNase H cleavage rate of hybrid duplexes
Source: Nucleic Acids Res. 2014 Feb 12;42(8):5378–89. doi: 10.1093/nar/gku125 (PMC4005664; doi:10.1093/nar/gku125)
Supplement: Supplementary Data [file supp_gku125_nar-02654-f-2013-File002.pdf]

Supplementary materials

SPR measurements

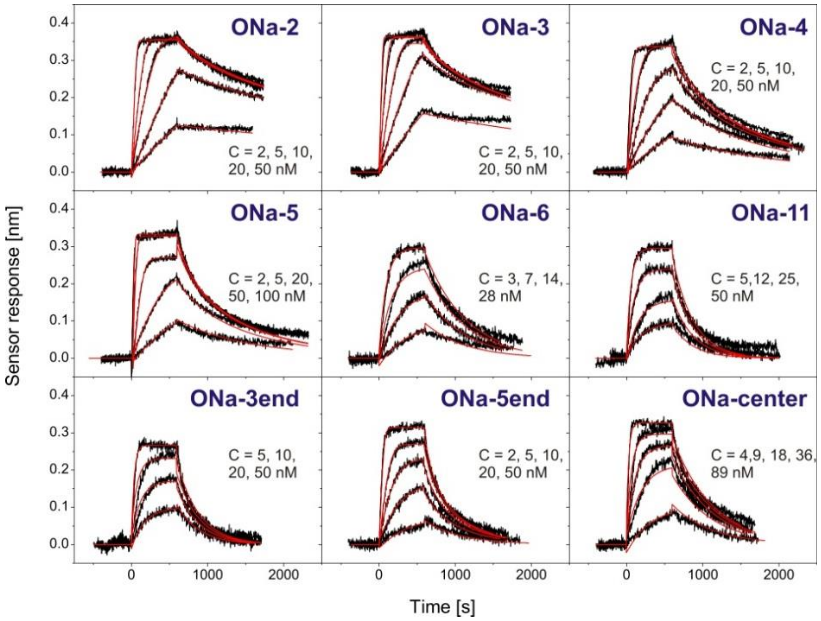

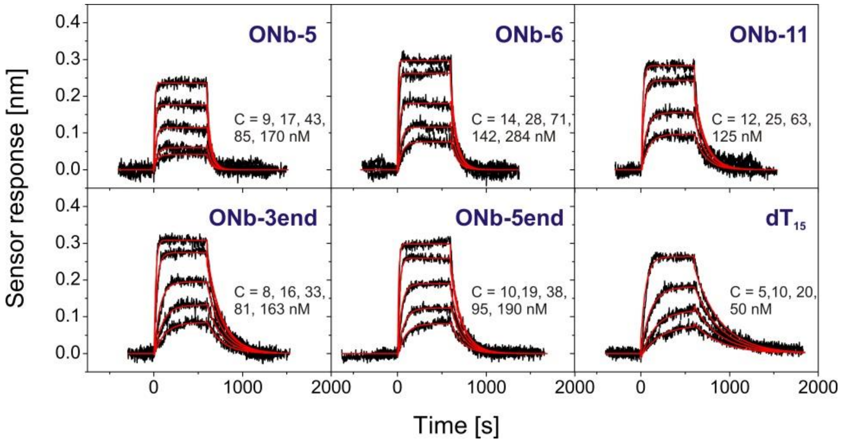

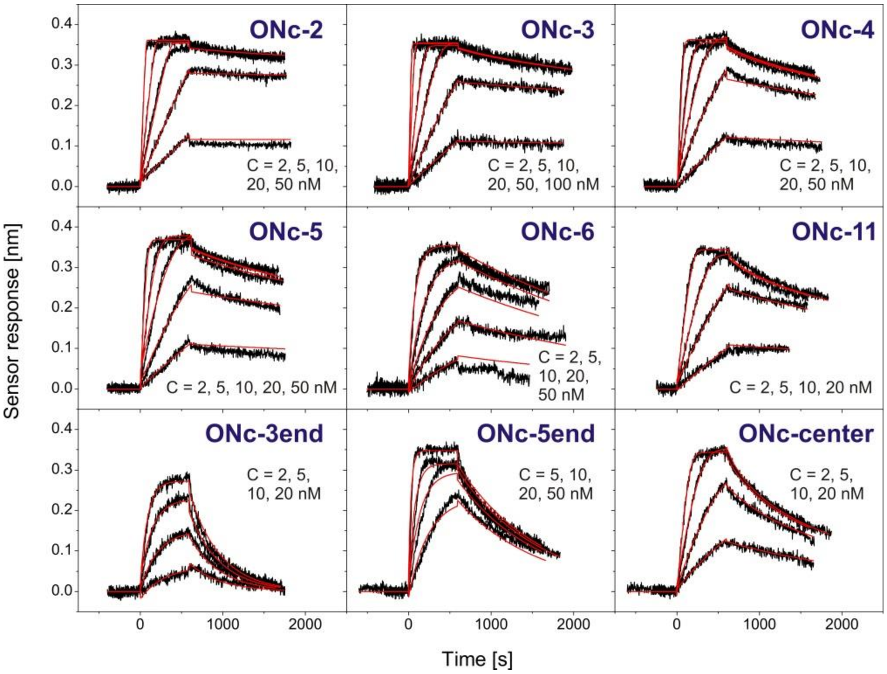

Figure S1: *SPR sensor response to hybridization of modified oligothymidylates at various concentrations to immobilized P1 (black curves). Each set of concentrations was fitted with Langmuir 1:1 model in BIAevaluation software to obtain affinity rate constants (red curves).*

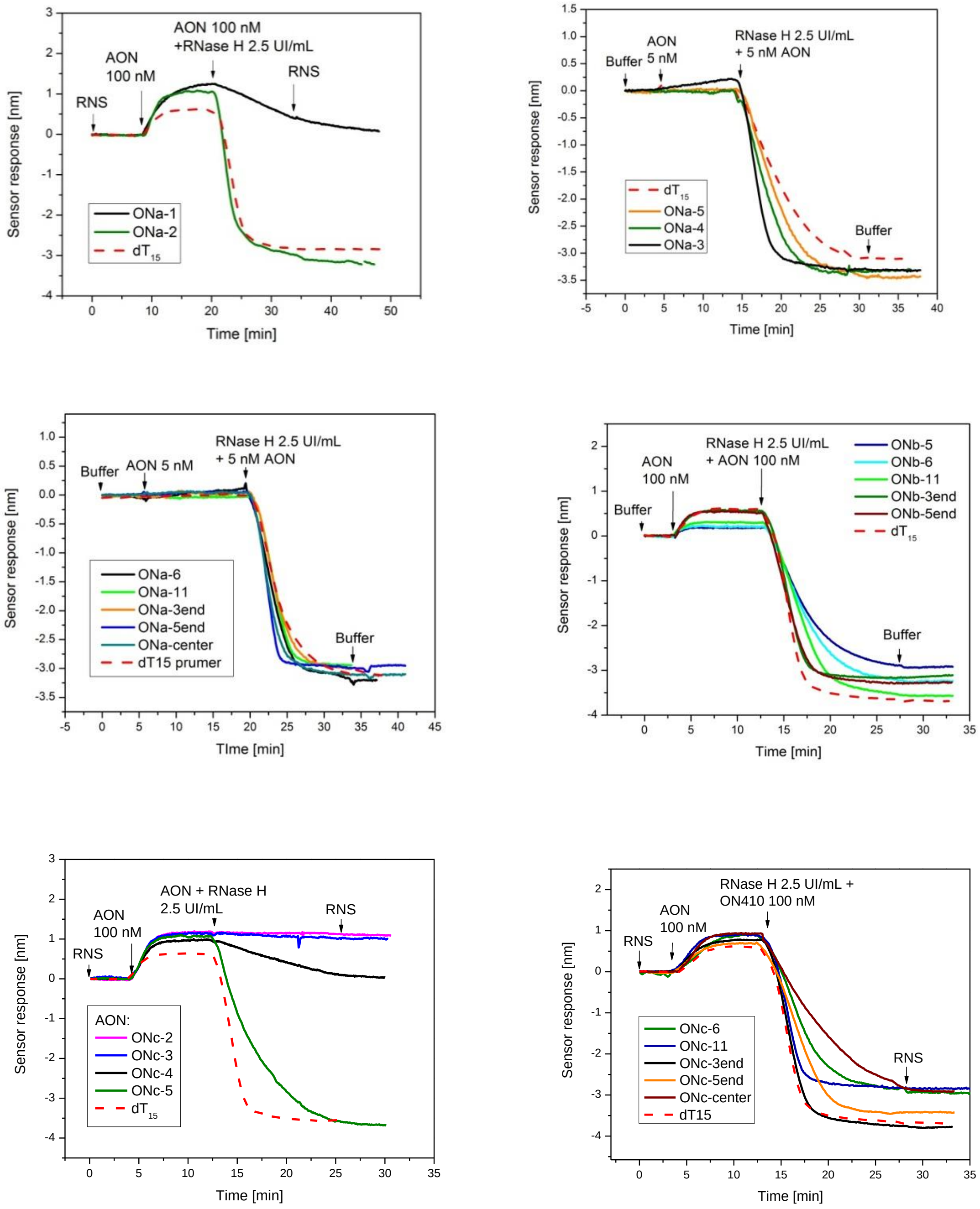

Figure S2: *SPR sensor response to hybridization of modified oligothymidylates at various concentrations to immobilized P1 and hydrolysis of P1 by RNase H. Arrows indicate injection of the respective solutions.*

HPLC analysis of RNase H cleavage rate

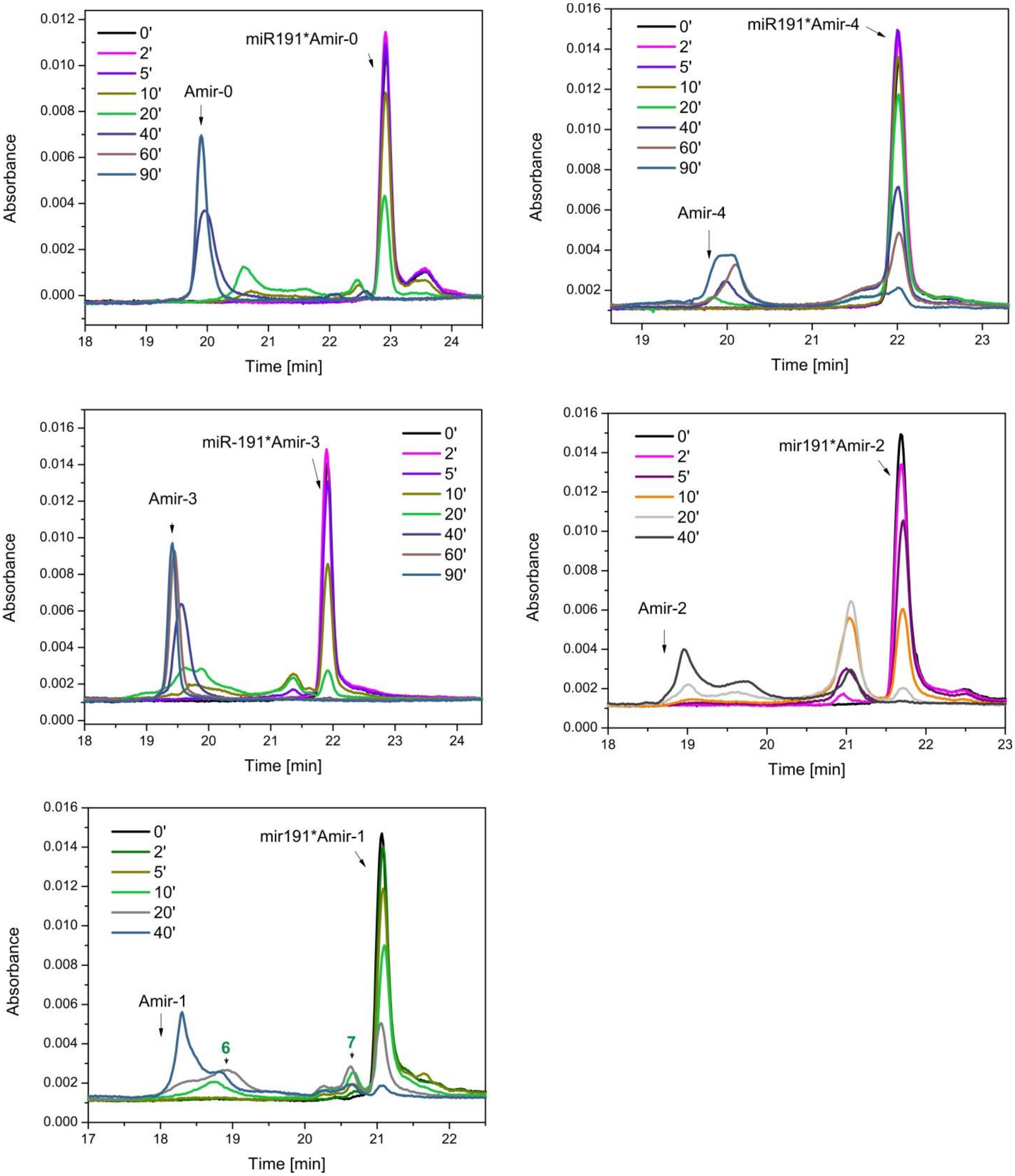

Figure S3: *HPLC separation of products of miR191 cleavage by RNase H E. coli, which was stopped in various times. Section of chromatogram shows miR191*Amir duplexes, Amir and in between the Amir hybridized with fragments of miR191. The enzymatic reaction was stopped in given times by addition of EDTA to the solution.*

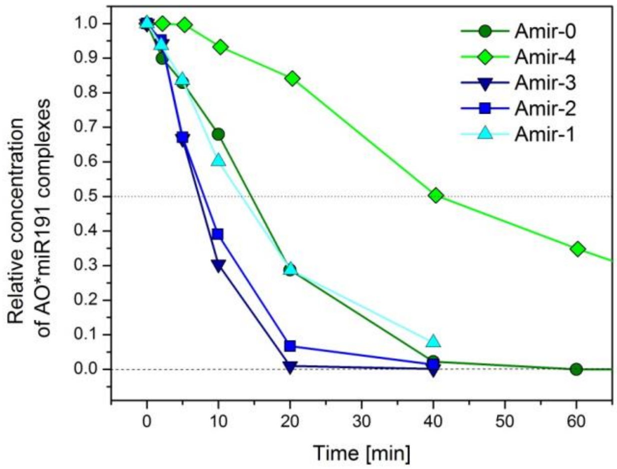

Figure S4: *Kinetics of RNase H cleavage of miRNA191 determined with HPLC. Area under peak corresponding to miR191*Amir hybrid duplex (Figure S3) was integrated and normalized to its area at time 0.*

Molecular dynamics simulations

MD simulations were produced using NVIDIA graphical processing units. The AMBER force field (46,47) was used for biomolecules surrounded by TIP3P water molecules (48).

MD trajectories (lasting for 50/100 ns at 310 K) for complexes of *E. coli* RNase H and $rA_{10}.dT_{10}$ were produced using the NAMD 2.7 software package (49). The dT_{10} was either natural, or it contained phosphodiester linkages alternating with 3'/5'-O-methylphosphonate, 5'(S)-hydroxyphosphonate or 2'-O-methyl internucleotide linkages. The smooth Particle-mesh Ewald (PME) method was employed for long-range electrostatic forces (50). The non-bonded cutoff was set to 9 Å. The SHAKE algorithm (tolerance 0.0005) was applied to constrain bonds where the hydrogen atoms were involved (51). After reaching the energy minimum of simulated systems the Langevin dynamics was used for a temperature control (49). The Langevin piston method was applied to reach an efficient pressure control with target pressure set to 1 atm (49). The integration time step was set to 2 fs.

The ACEMD software package (52,53) was used for production MD trajectories (lasting for 100 ns at 350K) of complexes of *E. coli* RNase H and $rA_{10}.dT_{10}$ as well as of both MD simulations (lasting for 1000 ns at 400K) of $dT_{12}.dA_{12}*dT_{12}$ triple helical structures with alternating natural and 5'(R/S)-hydroxyphosphonate internucleotide linkages in both dT_{12} strands. ACEMD is molecular dynamics software of new generation, which runs exclusively on GPUs at the equivalent speed of tens to hundreds of standard processors. ACEMD implements all features of an MD simulation on a CUDA-compatible GPU device, including those usually required for production simulations in the NVT ensemble (i.e. isothermal, isochoric), which include bonded and nonbonded force term computation, velocity-Verlet integration, Langevin thermostatic control, smooth PME, and hydrogen bond constraints implemented using the M-shake algorithm and RATTLE for velocity constraints within the velocity Verlet integration scheme (54,55). At present, ACEMD does not contain a barostat for production runs, so simulations in the NPT (i.e. isothermal, isobaric) ensemble are not possible. However, it is noted that with large

molecular systems, changes in volume due to the pressure control are very limited after an initial equilibration making NVT simulations viable for production runs (52,53). It was proven on multiple biomolecular systems including membrane proteins (56-58). Therefore, our production runs have been performed in the NVT ensemble using the Langevin thermostat. Electrostatic interactions were summed with the PME method. Also implemented in ACEMD is the hydrogen mass repartitioning scheme (the mass of the bonded heavy atoms to hydrogen is repartitioned among hydrogen atoms, leaving the total mass of the system unchanged) used, for instance, in codes such as Gromacs, which allows an increased time step of up to 4 fs (52,59), which was set in our production MD runs. MD trajectories were analyzed with the aid of the VMD 1.9 and AMBER10/ptraj software packages (60,61). Figures were produced by means of the ICM Molsoft 3.7 software package.

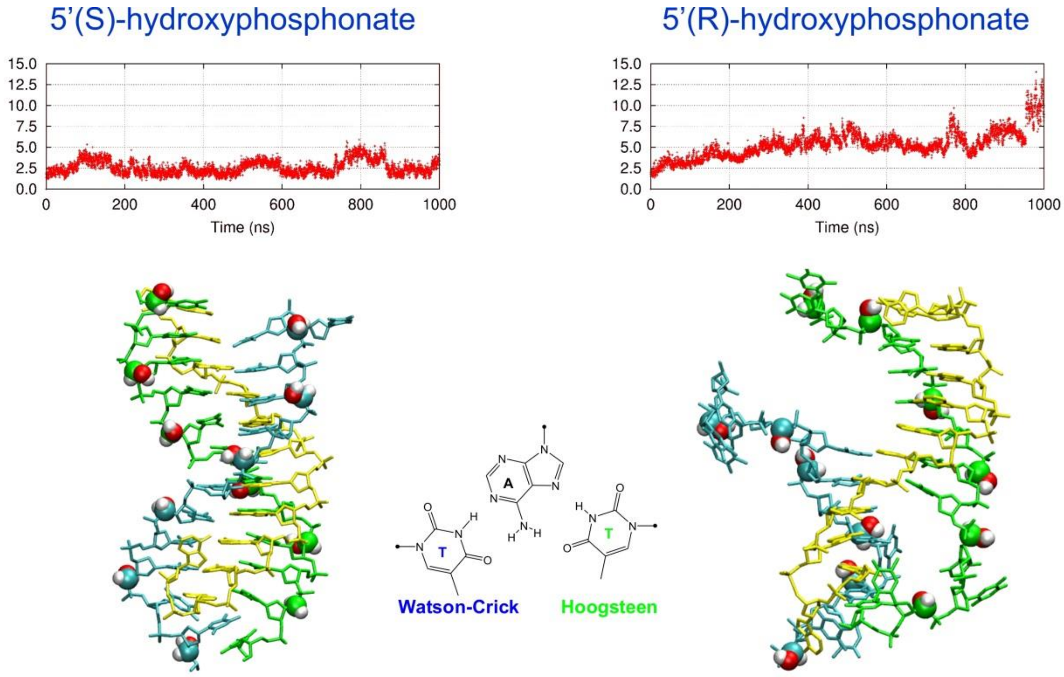

Figure S5. *Results of MDS (1000 ns at 400 K temperature) testing the stability of triple helical structures consisting of the central dA12 strand (yellow) and its Watson-Crick (cyan) and Hoogsteen (green) counterparts, both deoxythymidine dodecamers with alternating the natural phosphate and the modified 5'-hydroxyphosphonate linkages. The left half of the figure concerns the 5'(S) type of the hydroxyphosphonate linkages and the right part the 5'(R) type of the modified linkage. Time evolutions of the Root-Mean-Square Deviation values (RMSD) on the top shows remarkable stability of the 5'(S)-hydroxyphosphonate triplex in contrast to that with 5'(R)-hydroxyphosphonate linkages, which disintegrated completely. Obtained time developments of hydrogen bonds and torsion angles are shown in Figure S6 for the 5'(S) type and in Figure S7 for the 5'(R) type.*

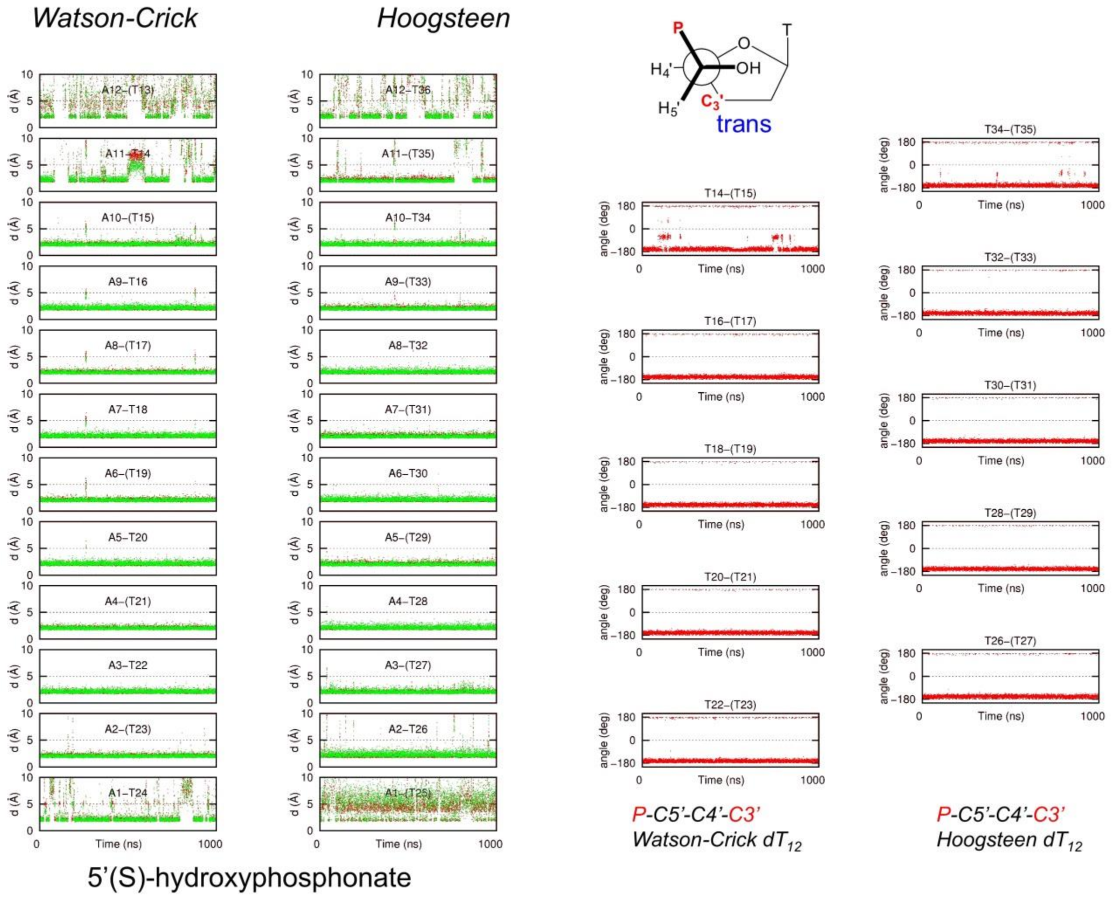

Figure S6. *Time developments of particular structural parameters obtained during 1000 ns MDS run at 400K for the stable triple helical structure (see Figure S5, left) carrying the 5'(S)-hydroxyphosphonate internucleotide linkages. Left: Time developments of the two (distinguished by red and green color) Watson-Crick and two Hoogsteen hydrogen bonds in individual base triplets. Hydrogen bonds were stable except for the terminal triplets. Right: Time developments of P-C5'-C4'-C3' torsion angles in 5'(S) hydroxyphosphonate internucleotide linkages. Strong preference for the trans conformer is evident for all linkages.*

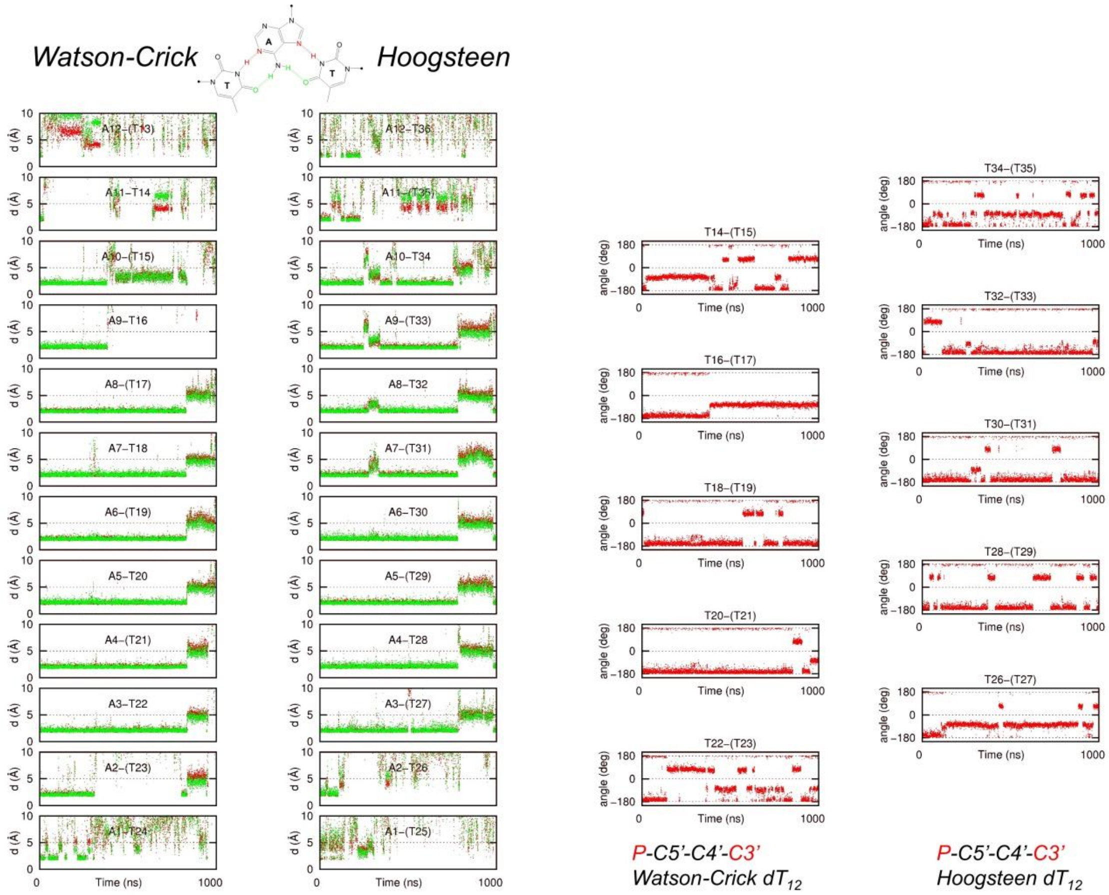

Figure S7. *Time developments of particular structural parameters obtained during 1000 ns MDS run at 400K for the unstable triple helical structure (see Figure S5, right) carrying the 5'(R)-hydroxyphosphonate internucleotide linkages. Left: Time developments of the two (distinguished by red and green color) Watson-Crick and two Hoogsteen hydrogen bonds in individual base triplets. Hydrogen bonds in the first and the last couple of triplets were unstable from the beginning of the run, at the end of the run the triplet structure is disintegrated completely. Right: Time developments of* ***P****-C5'-C4'-****C3****' torsion angles in 5'(R)-hydroxyphosphonate internucleotide linkages. The* ***P****-C5'-C4'-****C3****' torsion angle is conformationally promiscuous passing between* ***trans****,* ***+gauche*** *and* ***-gauche*** *conformers.*

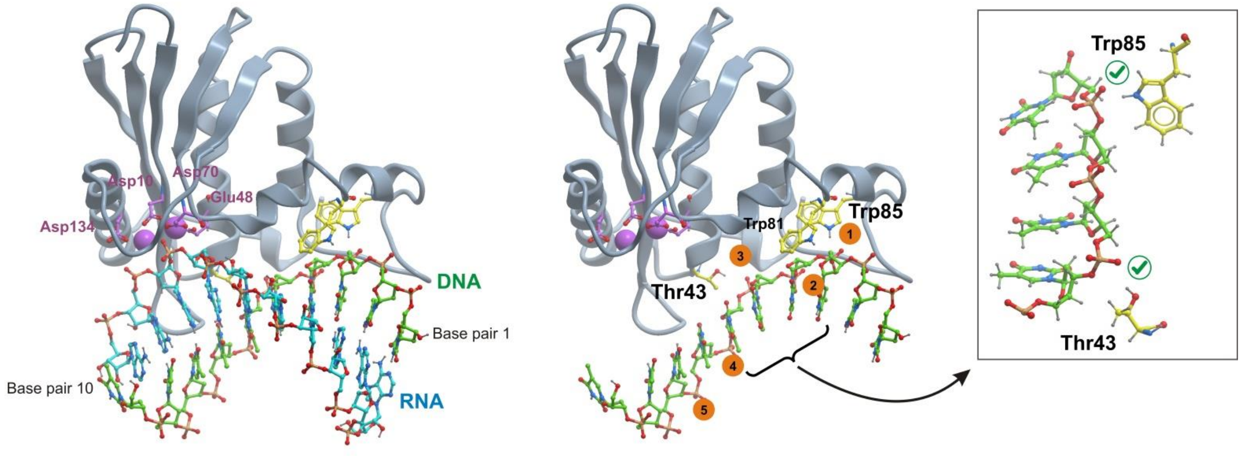

Figure S8. *Left: Complex consisting of E. coli RNase H and natural* rA_{10}*:*dT_{10} *hybrid duplex. The sticks depict, besides DNA (green) and RNA (blue) strands, also the side chains of amino acids stabilizing two divalent magnesium ions in the E. coli RNase H active site (violet) and the side chains of Trp85, Trp81 and Thr43 amino acids forming DNA binding sites (yellow). Magnesium ions (violet spheres) interact with the scissile internucleotide linkage of RNA. Right: The complex without shown RNA strand. Five internucleotide linkages in DNA reaching from Trp85 to the position opposite to the scissile phosphate of RNA (marked by numbers) seem to be the most critical for E. coli RNase H -* dT_{10} *recognition. The inset shows detail view of hydrogen bonding between phosphate groups of DNA and Trp85 / Thr43 amino acids.*

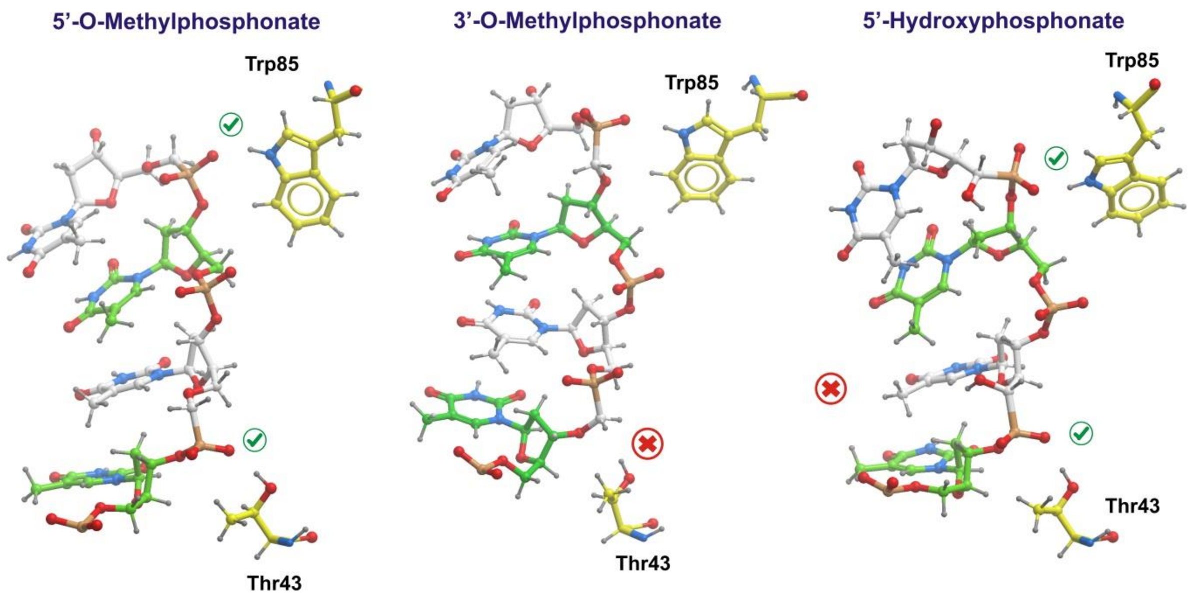

Figure S9. *Detail view of interactions between phosphonate groups of modified DNA chains (with alternating natural and modified linkages) and Trp85 / Thr43 amino acids in DNA binding sites of E. coli RNase H. Left: The 5'-O-methylphosphonate internucleotide linkages are able to interact potently with both amino acids side chains. Center: In contrast, the methylene group positioning in the 3'-O-methylphosphonate internucleotide linkage disables completely the hydrogen bonding with Thr43. Moreover, contacts with Trp85 disappeared within MD runs (see Figure S10 and S11). Right: The 5'(S)-hydroxyphosphonate internucleotide linkages were able to interact efficiently with both amino acids side chains. Nevertheless, it ultimately led to disruption of Watson-Crick hydrogen bonds (see Figure 10).*

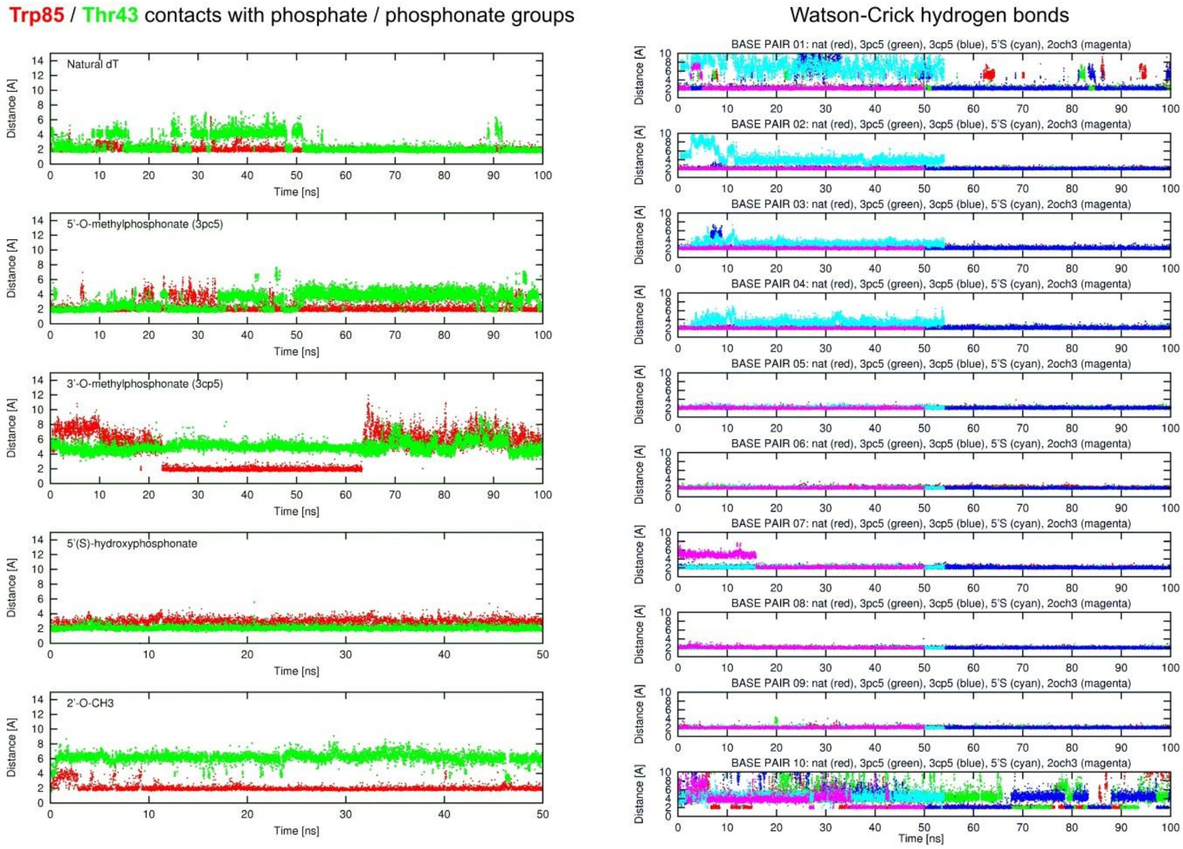

Figure S10. *Left: Time developments of hydrogen bonds between phosphate/phosphonate internucleotide linkages of DNA and Trp85 (red) and Thr43 (green) side chains in DNA binding sites in the complex consisting of E. coli RNase H and a hybrid duplex formed by rA_{10} and dT_{10} analogs with alternating natural and modified (as indicated) linkages. 50/100 ns MD trajectories at 310 K. The natural and 5'-O-methylphosphonate internucleotide linkages are able to interact potently with both amino acids side chains. In contrast, the methylene group in the 3'-O-methylphosphonate internucleotide linkage obstructs completely hydrogen bonding with Thr43. Moreover, contacts with Trp85 disappeared within a MD run. The 5'-(S)-hydroxyphosphonate internucleotide linkages were able to interact efficiently with both amino acids side chains. Nevertheless, it ultimately led to disruption of some Watson-Crick hydrogen bonds. The 2'-O-methyl group causes steric conflicts with the E. coli RNase H surface. It hinders hydrogen bonding with Thr43. Right: Time development of the Watson-Crick hydrogen bondings in individual base pairs of the hybrid duplex. Note disturbances in the case of the 5'(S)-hydroxyphosphonate modification (cyan).*

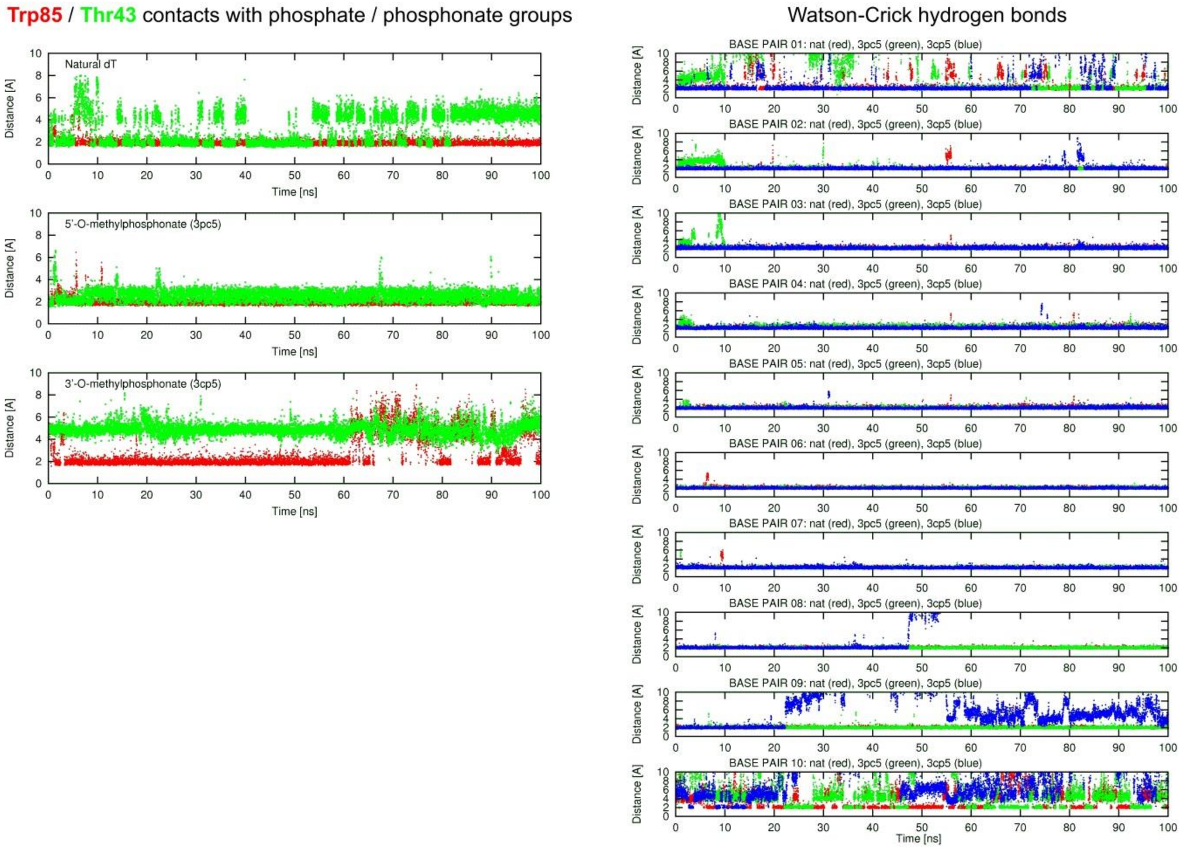

Figure S11. *Time developments obtained by 100ns MD runs of the same systems as in the case of Figure S10 but at temperature of 350K. Left: The natural and 5'-O-methylphosphonate internucleotide linkages are able to interact potently with both amino acids side chains. In contrast, the methylene group positioning in the 3'-O-methylphosphonate internucleotide linkage prevents hydrogen bonding with Thr43. Moreover, contacts with Trp85 disappeared within a MD run. Right: Note disturbances in the case of 3'-O-methylphosphonate-dT_{10}.rA_{10} (blue).*

References

46. Cornell, W.D., Cieplak, P., Bayly, C.I., Gould, I.R., Merz, K.M., Ferguson, D.M., Spellmeyer, D.C., Fox, T., Caldwell, J.W. and Kollman, P.A. (1995) A 2nd Generation Force-Field for the Simulation of Proteins, Nucleic-Acids, and Organic-Molecules. *J Am Chem Soc*, **117**, 5179-5197.
47. Perez, A., Marchan, I., Svozil, D., Sponer, J., Cheatham, T.E., Laughton, C.A. and Orozco, M. (2007) Refinenement of the AMBER force field for nucleic acids: Improving the description of alpha/gamma conformers. *Biophys J*, **92**, 3817-3829.
48. Jorgensen, W.L., Chandrasekhar, J., Madura, J.D., Impey, R.W. and Klein, M.L. (1983) Comparison of Simple Potential Functions for Simulating Liquid Water. *J Chem Phys*, **79**, 926-935.
49. Phillips, J.C., Braun, R., Wang, W., Gumbart, J., Tajkhorshid, E., Villa, E., Chipot, C., Skeel, R.D., Kale, L. and Schulten, K. (2005) Scalable molecular dynamics with NAMD. *J Comput Chem*, **26**, 1781-1802.

50. Cheatham, T.E., Miller, J.L., Fox, T., Darden, T.A. and Kollman, P.A. (1995) Molecular-Dynamics Simulations on Solvated Biomolecular Systems - the Particle Mesh Ewald Method Leads to Stable Trajectories of DNA, Rna, and Proteins. *J Am Chem Soc*, **117**, 4193-4194.
51. Ryckaert, J.P., Ciccotti, G. and Berendsen, H.J.C. (1977) Numerical-Integration of Cartesian Equations of Motion of a System with Constraints - Molecular-Dynamics of N-Alkanes. *J Comput Phys*, **23**, 327-341.
52. Harvey, M.J., Giupponi, G. and De Fabritiis, G. (2009) ACEMD: Accelerating Biomolecular Dynamics in the Microsecond Time Scale. *J Chem Theory Comput*, **5**, 1632-1639.
53. , Vol. 2013.
54. Andersen, H.C. (1983) Rattle - a Velocity Version of the Shake Algorithm for Molecular-Dynamics Calculations. *J Comput Phys*, **52**, 24-34.
55. Lambrakos, S.G., Boris, J.P., Oran, E.S., Chandrasekhar, I. and Nagumo, M. (1989) A Modified Shake Algorithm for Maintaining Rigid Bonds in Molecular-Dynamics Simulations of Large Molecules. *J Comput Phys*, **85**, 473-486.
56. Giorgino, T. and De Fabritiis, G. (2011) A High-Throughput Steered Molecular Dynamics Study on the Free Energy Profile of Ion Permeation through Gramicidin A. *J Chem Theory Comput*, **7**, 1943-1950.
57. Selent, J., Sanz, F., Pastor, M. and De Fabritiis, G. (2010) Induced Effects of Sodium Ions on Dopaminergic G-Protein Coupled Receptors. *Plos Comput Biol*, **6**.
58. Buch, I., Giorgino, T. and De Fabritiis, G. (2011) Complete reconstruction of an enzyme-inhibitor binding process by molecular dynamics simulations. *Proceedings of the National Academy of Sciences of the United States of America*, **108**, 10184-10189.
59. Feenstra, K.A., Hess, B. and Berendsen, H.J.C. (1999) Improving efficiency of large time-scale molecular dynamics simulations of hydrogen-rich systems. *J Comput Chem*, **20**, 786-798.
60. Humphrey, W., Dalke, A. and Schulten, K. (1996) VMD: Visual molecular dynamics. *J Mol Graph Model*, **14**, 33-38.
61. Pearlman, D.A., Case, D.A., Caldwell, J.W., Ross, W.S., Cheatham, T.E., Debolt, S., Ferguson, D., Seibel, G. and Kollman, P. (1995) Amber, a Package of Computer-Programs for Applying Molecular Mechanics, Normal-Mode Analysis, Molecular-Dynamics and Free-Energy Calculations to Simulate the Structural and Energetic Properties of Molecules. *Comput Phys Commun*, **91**, 1-41.
